# *Natterin-like* depletion by CRISPR/Cas9 impairs zebrafish (*Danio rerio*) embryonic development

**DOI:** 10.1186/s12864-022-08369-z

**Published:** 2022-02-12

**Authors:** Ana Carolina Seni-Silva, Adolfo Luis Almeida Maleski, Milena Marcolino Souza, Maria Alice Pimentel Falcao, Geonildo Rodrigo Disner, Monica Lopes-Ferreira, Carla Lima

**Affiliations:** 1 Immunoregulation Unit of the Laboratory of Applied Toxinology (CeTICs/FAPESP), Butantan Institute, Vital Brazil Avenue, 1500, Butantan, São Paulo 05503-009 Brazil; 2 Post-Graduation Program of Toxinology, Butantan Institute, São Paulo, SP Brazil

**Keywords:** Natterin proteins family, *Danio rerio*, Embryogenesis, CRISPR/Cas9, Phenotype-based screening, Physiological functions

## Abstract

**Background:**

The Natterin protein family was first discovered in the venom of the medically significant fish *Thalassophryne nattereri*, and over the last decade *natterin-like* genes have been identified in various organisms, notably performing immune-related functions. Previous findings support *natterin-like* genes as effector defense molecules able to activate multiprotein complexes driving the host innate immune response, notably due to the pore-forming function of the aerolysin superfamily members. Herein, employing a combination of the CRISPR/Cas9 depletion system, phenotype-based screening, and morphometric methods, we evaluated the role of one family member, *LOC795232*, in the embryonic development of zebrafish since it might be implicated in multiple roles and characterization of the null mutant is central for analysis of gene activity.

**Results:**

Multiple sequence alignment revealed that the candidate *natterin-like* has the highest similarity to zebrafish *aep1*, a putative and better characterized fish-specific defense molecule from the same family. Compared to other species, zebrafish have many *natterin-like* copies. Whole-mount in situ hybridization confirmed the knockout and mutant embryos exhibited epiboly delay, growth retardation, yolk sac and heart edema, absent or diminished swim bladder, spinal defects, small eyes and head, heart dysfunction, and behavioral impairment. As previously demonstrated, ribonucleoproteins composed of Cas9 and duplex guide RNAs are effective at inducing mutations in the F0 zebrafish.

**Conclusions:**

The considerably high *natterin-like* copies in zebrafish compared to other species might be due to the teleost-specific whole genome duplication and followed by subfunctionalization or neofunctionalization. In the present work, we described some of the *natterin-like* features in the zebrafish development and infer that natterin-like proteins potentially contribute to the embryonary development and immune response.

**Supplementary Information:**

The online version contains supplementary material available at 10.1186/s12864-022-08369-z.

## Highlights

- The Natterin family was discovered in the venom of the fish *Thalassophryne nattereri.*
- The zebrafish genome encodes eleven *natterin-like* genes.
- *Natterin-like* might be a novel fish-specific defense molecule.
- Natterin-like proteins are thought to be pore-forming molecules.
- Reverse genetic study and phenotypic characterization suggests *natterin-like* genes may have roles in zebrafish development.

**Supplementary Information:**

The online version contains supplementary material available at 10.1186/s12864-022-08369-z.

## Background

Over the past 15 years, significant progress has been made in expanding the knowledge on the Natterin family since the first discovery of the four founder members in the venom of the medically significant toadfish *Thalassophryne nattereri* (V*Tn*) [1]. One of the main symptoms resulting from *T. nattereri* envenomation is the immediate and intense pain that persists over 24 h. Erythema and edema are also shortly noticed with the efflorescence of bubbles with serous content. These lesions progress to long-remaining necrosis with a delayed healing process [2].

The presence of Natterin family members in evolutionarily divergent non-venomous species suggests an adaptive value consistent with the functions plurality, including immunity, signaling, and development rather than its feature only as a toxin. It is believed that Natterins have a substantial role as effector defense molecules. Their ability to activate cells and cytoplasmic multiprotein complexes that drive the host innate immune response might be due to the fact that all Natterin-like proteins have pore-forming domains and are generally classified as part of the aerolysin superfamily [3–7].

Moreover, the *natterin-like* genes have been identified as constitutively expressed in various immune-related tissues (e.g., hemolymph, blood, kidney, spleen, gills) as well as in the heart, liver, and gonads [8–14]. Their expression is significantly regulated upon parasite, bacterial and viral infections, and even by abiotic stressors in species such as the Atlantic salmon (*Salmo salar*) [8], lamprey (*Lampetra morii*) [9, 10], common carp (*Cyprinus carpio*) [11], Atlantic cod (*Gadus morhua*) [12], zebrafish (*Danio rerio*) [13], and zebra mussel (*Dreissena polymorpha*) [14]. It was demonstrated by Chen et al. [15] that the pre-injection of recombinant Natterin-like (encoded by *aep1*, formerly *dln1*) into the infected zebrafish dramatically decreased the expression level of cytokines and accelerated the clearance of bacteria, resulting in significantly increased survival rate.

The *aep1,* the best-known natterin-like protein from zebrafish, was suggested as a novel defense molecule and the first one to have its structures unveiled by crystal and electron microscopy [13], along with additional functions reported in other models; then it has been used as a reference to compare with other zebrafish natterin-like. Considering that, to expand the knowledge over this protein family, we decided to focus on another zebrafish protein that lacks functional description in the literature and has the highest sequence similarity to *aep1*, i.e., *LOC795232*.

During the last three decades, zebrafish has emerged as an alternative vertebrate model for molecular and cellular development studies. Compared with other traditional vertebrate models, it offers advantages like a high reproduction rate, easy use and low cost, and high homology of genes known to be associated with human diseases. The embryo transparency and the easy prospect for genetic edition, including the combined system CRISPR (clustered regularly interspaced short palindromic repeats) with Cas9 nuclease [16], makes it possible for a wide exploration of the molecular mechanisms involved in different physiological and developmental processes.

Together with the discovery of the wide expression of *natterin-like* in different organisms, these advances provide a broader perspective of mechanisms by which pathways featuring *natterin-like* may protect against infections and provide specificity in physiological processes. Therefore, we hypothesize that in addition to Natterin playing a role as toxins and as effector molecule in immunity, it might have a role in fish development as other examples of critical genes mentioned in the literature that also have multiple roles, notably in immune response and development, such as β-defensins and HOX, respectively [17, 18].

To test the hypothesis that members of the Natterin family act in key functions, we evaluate in zebrafish a combination of RNA duplexes of chemically synthesized crRNA and trans-activating crRNA (tracrRNA) molecules complexed with Cas9 protein to form duplex guide ribonucleoproteins (dgRNPs) that act as a depletion system. Phenotype-based screening and morphometric methods were applied to evaluate the specific function of the *natterin-like* LOC795232, present on zebrafish chromosome 7, in the early embryonic stages of development.

## Results

### Multiple alignment analysis of *Natterin-like* in zebrafish

Many genes were duplicated during teleost evolution after divergence from the tetrapod lineage [19]. In *T. nattereri* four genes are responsible for encoding all members of the Natterin family (natterin 1 to 4). A bioinformatic survey aiming to know how many natterin genes are in zebrafish confirmed that its genome contains 10 *natterin-like* genes encoding 11 proteins as described in Table 1. When compared in terms of structure, all of them have an aerolysin-like motif in the C-terminal region (~ 116–360 aa) and a jacalin-like module in the N-terminal region (~ 13–186 aa), except XP_021325134.1, the largest natterin-like protein of zebrafish with 368 amino acids (aa), encoded by *LOC101882550,* which has the aerolysin-like domain in C-terminal and unknown module in N-terminal portion.

**Fig. 1:**
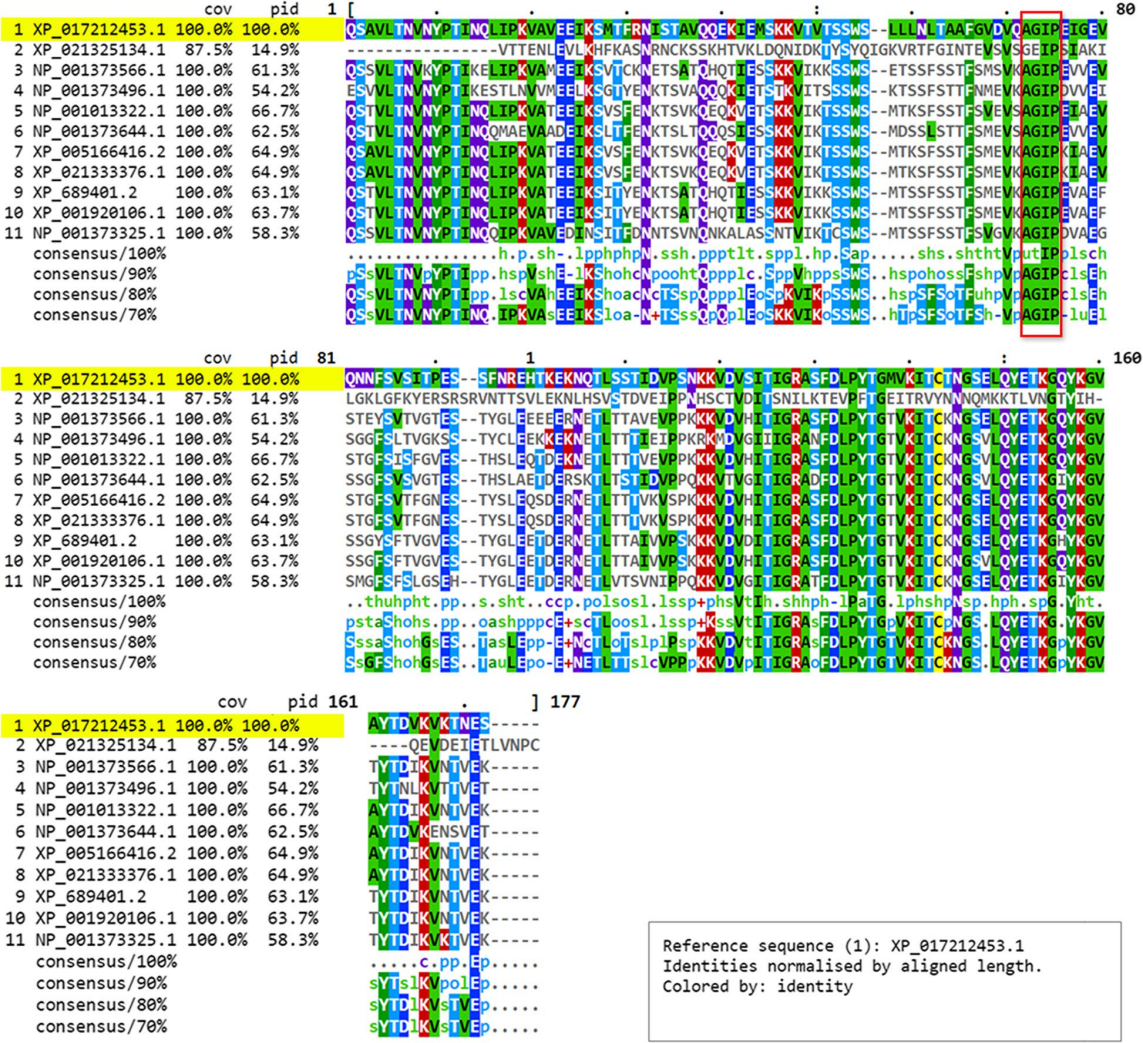
Multiple sequence alignment of the zebrafish natterin-like proteins evidencing the identity among residues within the aerolysin domain. The zebrafish natterin-like protein sequences were obtained from National Center for Biotechnology Information (NCBI) and aligned through Clustal Omega (European Molecular Biology Laboratory - EMBL/European Bioinformatics Institute - EBI). The alignment is displayed in MView evidencing the coverage (cov), percentage of identity (pid), and consensus among proteins. The highlight in yellow→indicates the reference natterin protein studied herein coded by *LOC795232* and the Natterin family conserved residues (AGIP) are highlighted in the red square

The comparison among zebrafish natterin-like proteins supported the selection of the XP_017212453.1 encoded by *LOC795232* as the candidate of our study since it presents the highest identity with well-known similar proteins. All zebrafish natterin-like [except XP_021325134.1, percentage of identity (PID) 10.1%] had a PID ranging from 50.6 to 60.6% with the candidate gene (Table 1). When the sequences were compared in the conserved β portion of the aerolysin domain, the similarity to our subject increased and ranged from 54.2 to 66.7%. In contrast, the NP_001013322.1 protein, encoded by another putative zebrafish *natterin-like* (*aep1*), showed the higher identity for either the complete sequence or conserved aerolysin domain comparison (Fig. 1, Table 1).

**Table 1:** Zebrafish (*Danio rerio*) natterin-like proteins

Zebrafish (*Danio rerio*)							
Gene (#10)	Isoform (#11)	Protein ID	Gene product	Aliases	Length (aa)	PID (%) Complete sequence	PID (%) Aerolysin domain
*LOC795232*	1	XP_017212453.1	Natterin-like protein	–	286	Ref.	Ref.
*aep1*	1	NP_001013322.1	Aerolysin-like/Natterin-like protein	*dl, dln1, jac, jac5, zgc:113413, zgc:174689*	315	60.6	66.7
*jac1*	2	XP_021333376.1	Jacalin 1/Natterin-like isoform 1	*–*	362	51.8	64.9
		XP_005166416.2	Jacalin 1/Natterin-like isoform 2	*fb11f10, si:dkeyp-32 g11.7, wu:fb11f10*	361	51.9	64.9
*jac2*	1	XP_689401.2	Jacalin 2/Natterin-like	*si:ch211-241c24.4*	315	59.1	63.1
*jac3*	1	XP_001920106.1	Jacalin 3/Natterin-like	*si:ch211-241c24.3*	315	59.1	63.7
*si:dkeyp-32 g11.8*	1	NP_001373644.1	si:dkeyp-32 g11.8/Natterin-like	*–*	315	58.1	62.5
*LOC5646602*	1	NP_001373566.1	Hypothetical protein LOC564660/ aerolysin-like/natterin-like	*–*	315	55	61.3
*jac4*	1	NP_001373325.1	Jacalin 4/Natterin-like	*fa92h10, si:dkeyp-32 g11.9, wu:fa92h10*	315	55.9	58.3
*LOC564481*	1	NP_001373496.1	hypothetical protein LOC564481/ aerolysin-like/natterin-like	*–*	313	50.6	54.2
*LOC101882550*	1	XP_021325134.1	Natterin-like 3	*–*	368	10.1	14.9

The zebrafish *natterin-like* sequences were obtained from National Center for Biotechnology Information (NCBI)

Then we found the conserved residues of the Natterin family, i.e., AGIP (ala-gly-ile-pro), in most zebrafish proteins, except XP_021325134.1. Unlike all four natterins found in *T. nattereri*, the zebrafish proteins also showed in the N-terminal region the YPT (tyr-pro-thr) conserved residues, a galactose-binding site [20], with a 90% consensus, and other conserved residues with unknown functions, like VLTNVN, FDLPYTG, AND LQYETKG_KGV, within the aerolysin domains, except for XP_021325134.1 (Fig. 1). These differences in the founder proteins from a venomous fish to those in zebrafish may highlight the variability in the function they exert in the latter, which seems to be more related to immune and developmental processes.

### *Natterin-like* gene LOC795232 is expressed during embryogenesis, and CRISPR/Cas9 successfully knocked it out

**Fig. 2:**
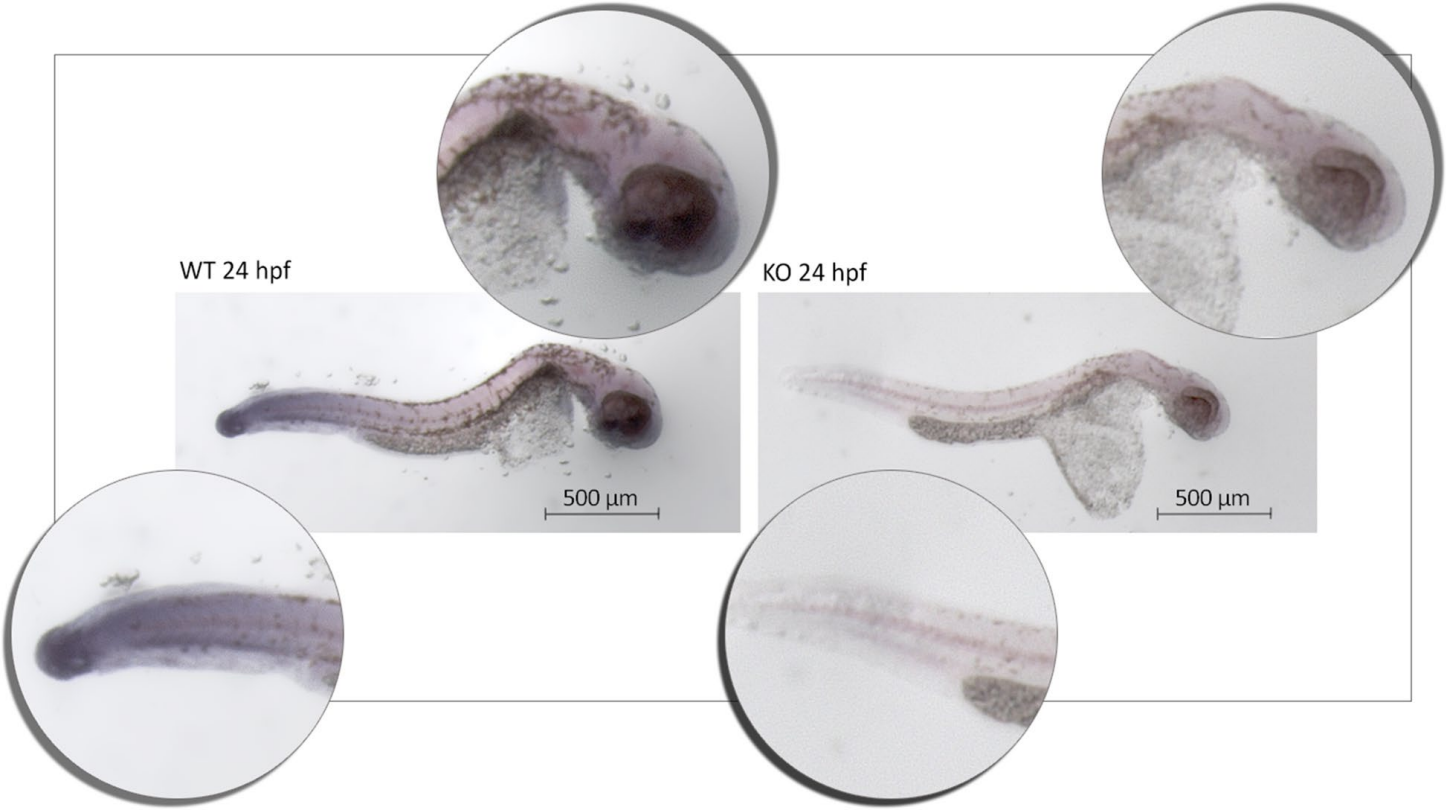
Qualitative evaluation of *natterin-like* gene expression by whole-mount in situ Hybridization (WISH). Larvae after 24 h post-fertilization (hpf) were fixed, dehydrated, permeabilized and hybridized with the DIG-labeled probe, complementing the natterin-like RNA sequence (Qiagen, #339500 LCD0168623-BKG). Probe hybridization was determined by binding the AP-labeled anti-DIG antibody (#11093274910, Roche Diagnostics) and revealed through chromogenic reaction by NBT/BCIP solution. The images were obtained in the stereomicroscope STEREO LUMAR-V-12 Carl Zeiss. The picture snippets indicate the area of the larva’s body where the blue-purple precipitate (indicating gene product) can be observed in the non-depleted wild-type (WT) larvae (left), indicative of the presence of the *natterin-like* transcripts; and in the depleted (KO) larvae by CRISPR/Cas9 system (crRNA natterin-like WD07479944, Sigma) (right). The embryos body changed the shape due to long-lasting high temperature incubation in conical-bottom microtubes, not representing developmental malformations

We followed the embryos from 24 to 144 h post-fertilization (hpf) to confirm the expression of *LOC795232* transcripts by qualitative whole-mount in situ hybridization (WISH) analysis, which is widely used for spatial and temporal detection of the expression throughout development [21]. The WISH suggests broadest expression at 24 hpf, and the transcripts were visualized in both the tailbud region and in the head, notably between the gill and heart regions of WT embryos, probably comprising the heart during the early stage of embryo development (24 to 144 hpf, Fig. 2). After this period, the expression decreased. However, from the hatching long-pec stage (48 hpf) to the early larval period (72 to 144 hpf), the *natterin-like* transcripts persisted at low levels restricted to the head region (data not shown). Further, WISH analysis of KO displayed a mild expression in the embryos compared to WT.

To investigate the *natterin-like* role in zebrafish development, we used CRISPR/Cas9 approach to generate a deletion using the synthesized duplex guide RNA (dgRNA) composed by crRNA and tracrRNA, identified according to Bae et al. [22], in exon 2 of chromosome 7 (genomic identity: ID 795232, XM_017356964, 1298 bp). The dgRNA-Cas RNP complex was microinjected in 0 hpf embryos as 50 ng.μL^− 1^ of dgRNA with Cas9 enzyme at 250 ng.μL^− 1^. In this experimental model, CRISPR/Cas9-mediated mutagenesis is considerably improved by using a two-RNA component (crRNA:tracrRNA) version of the CRISPR system and it had been shown previously to be effective at inducing mutations in zebrafish F0 embryos [23–25]. Considering the robustness of the system and validity in F0 embryos, no stable adult lines were generated in the study.

### *Natterin-like* gene LOC795232 loss promotes epiboly arrest and increased mortality and morphological abnormalities

We tested for the influence of *natterin-like* expression on development by analyzing epiboly, segmentation, survival, and occurrence of abnormalities. First, we assessed potential epiboly delay when expression was abolished in KO compared to WT, according to Bruce and Heisenberg [26]. A substantial delay in epiboly progression was observed in KO embryos from the oblong-sphere stage and throughout the doming and progression phase (Additional File 1).

**Fig. 3:**
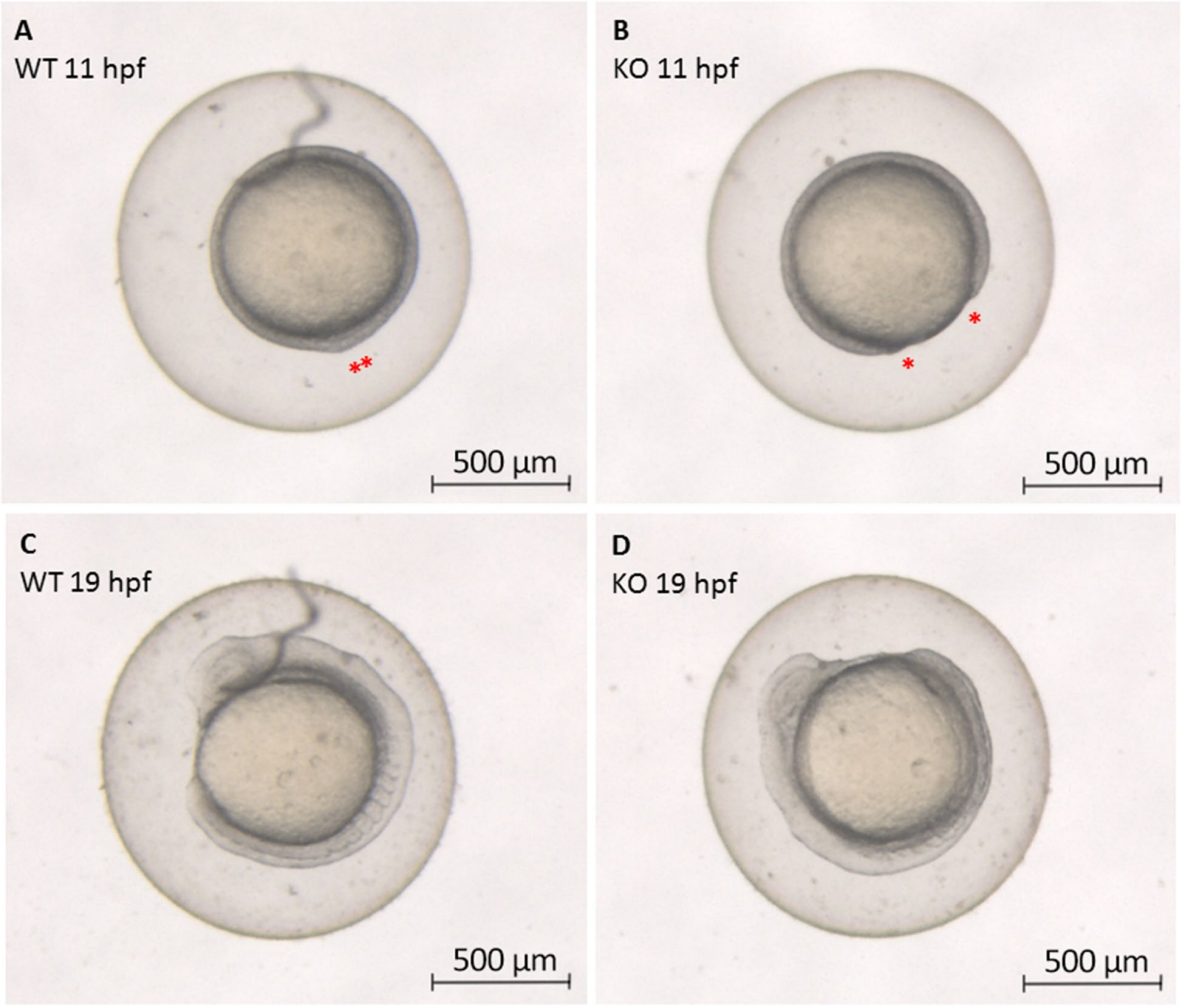
Zebrafish epiboly progression. The differences in epiboly progression are evidenced in wild-type (WT) (left) and CRISPR/Cas9 *natterin-like* KO (right) embryos at 11 and 19 h post-fertilization (hpf). The analysis was performed using a stereomicroscope Leica M205C (LAS V4.11 software) in a 27x magnification. The asterisks (*) represent the progress of blastopore until its closure at the end of gastrulation period. Representative figures from a sample of 20 embryos

Epiboly retard was first evident at 5 hpf. Then, at 11 hpf, we observed that KO embryos (Fig. 3B) did not present complete closure of the blastopore (red asterisks) at the end of the gastrulation period compared to WT embryos (Fig. 3A). At the segmentation period (19 hpf), when it was possible to observe anteroposteriorly elongated body with head, somites in the trunk, and tail and rudimentary organs in WT embryos (Fig. 3C), KO embryos (Fig. 3D) were indistinguishable in appearance, but the number of somites was lower than in WT.

**Fig. 4:**
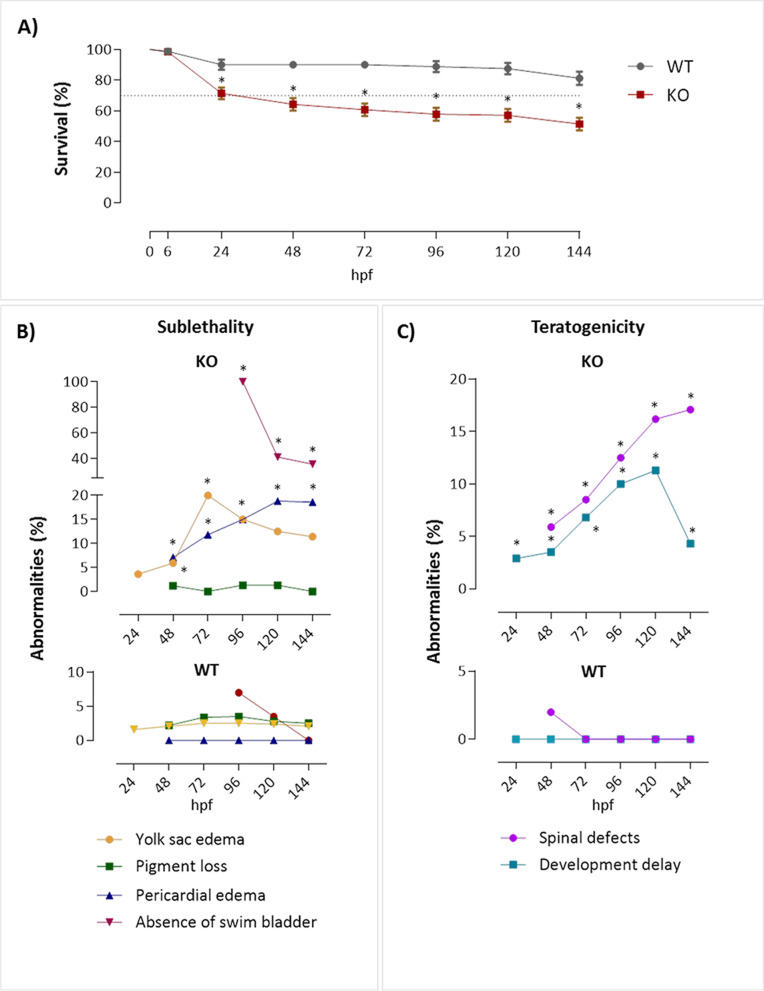
Survival rate, sublethal and teratogenic abnormalities of zebrafish after depletion of the *natterin-like* gene by the CRISPR/Cas9 system. The depletion of the *natterin-like* gene *LOC795232* in 0 h post-fertilization (hpf) zebrafish embryos was done by the synthetic duplex guide RNA (tracrRNA + natterin-like crRNA, 50 μg.nL^− 1^) with Cas9 (250 μg.nL^− 1^). The survival of the depleted larvae (KO) and wild-type (WT) group (**A**), sublethality (**B**), and teratogenicity (**C**) endpoints were analyzed at 24, 48, 72, 96, 120, and 144 hpf (20 embryos per group). The dashed line indicates the percentage of mortality acceptable by the OECD of 30%. The lines indicate the average of abnormalities in the KO group at each time measured. The asterisks (*) represent a significant difference with the WT (*p* < 0.05), which is represented in the complementary graph in the bottom of **B** and **C**. See additional file 2 for raw data of 20 embryos per group behind the figures

Moreover, we observed that KO embryos had a survival rate of 71.43% in the first 24 hpf compared to 90.12% in WT. The survival in mutants decreased over time, reaching 51.43% in 144 hpf, while the level of survival was 81.24% in WT (Fig. 4A). Next, we observed that KO showed yolk sac edema from 48 hpf (6%), reaching the highest percentage (20%) at 72 hpf (Fig. 4B). The KO abnormality incidence decreased by 96 hpf to 15% but remained high up to 120–144 hpf (12.5 and 11.4%, respectively). Still, delay in yolk absorption, which affects the efficiency of lipid metabolism, leading to a delay in the development of embryos is an example of sub-lethal abnormalities [27]. Regarding pericardial edema, the percentage of individuals with this abnormality progressively increased from 24 up to 120 hpf and stabilized at 144 hpf (7.1, 11.8, 15, 18.8, and 18.6%). As soon as the swim bladder became evident at 96 hpf [28], 100% of KOs did not present this organ. The frequency dropped to 41.3% at 120 hpf and 35.7% at 144 hpf, demonstrating a retard in its inflation. Still, Fig. 4B shows that few individuals (1.2% at 48 hpf, 1.3% at 96 hpf, and 1.3% at 120 hpf) had pigmentation deficiency (See additional files 2 and 3 for detailed phenotype-based screening data and supplemental images of embryos).

When teratogenic abnormalities were assessed in KOs, we observed increased frequency of individuals with spinal defects from 48 to 144 hpf (5.9, 8.5, 12.5, 16.2, and 17.1%). Moreover, from 24 to 120 hpf, we observed an increase in the developmental delay (2.9, 3.5, 6.8, 10, and 11.3%), which dropped to 4.3% at 144 hpf (Fig. 4C).

General morphological screening in early stages of development comprehended phenotypic measurements of the CRISPR/Cas9 system depletion efficacy. Alongside genotypic analysis, the phenotype evaluation makes it possible to screen the role of one or multiple genes [29], and the assessment can be reliably performed due to the extensive catalog of phenotypic changes in zebrafish described in the literature [30].

**Fig. 5:**
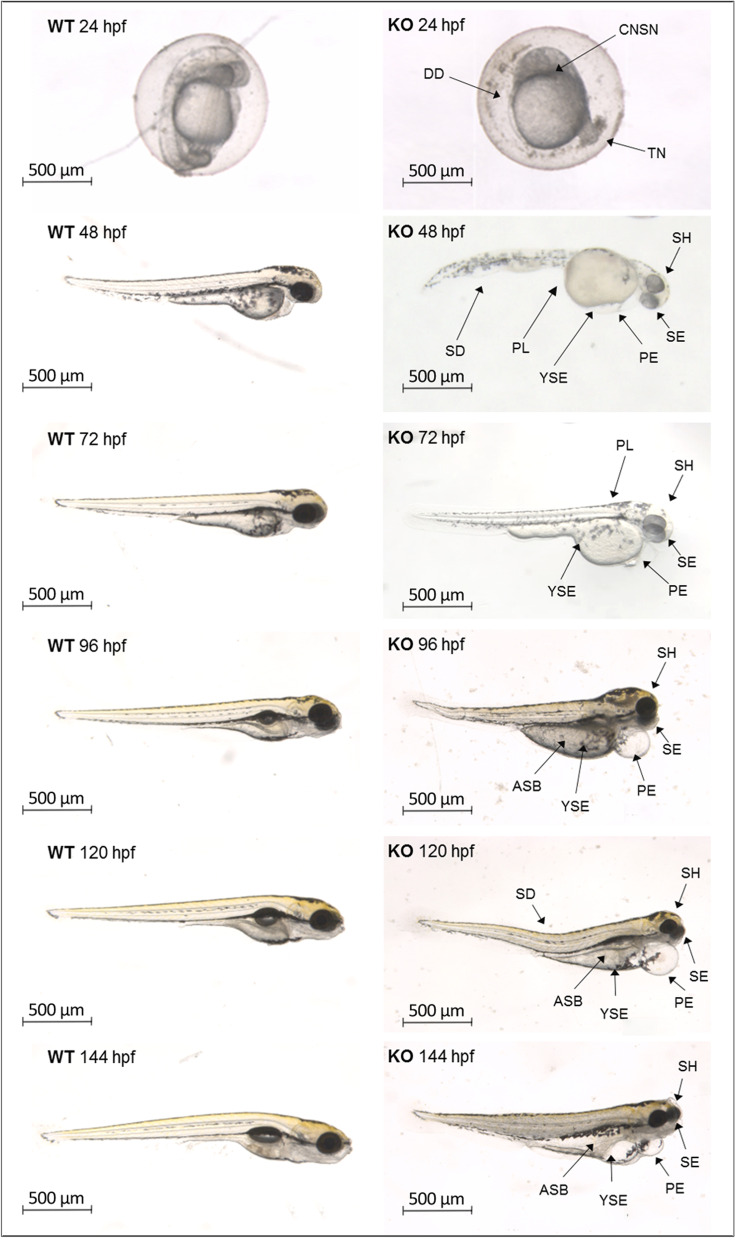
Analysis of aberrant phenotypes in *natterin-like* depleted (KO) zebrafish compared to wild-type (WT). The larvae where the *natterin-like* gene *LOC795232* was depleted (KO) by CRISPR/Cas9 system or WT were photographed in stereomicroscope Leica M205C (LAS V4.11 software, 27x magnification) at 24, 48, 72, 96, 120, and 144 h post-fertilization (hpf) (top to bottom) and analyzed for malformations: SD = spinal defect, YSE = yolk sac edema, PE = pericardial edema, ASB = absence of swimming bladder, DD = development delay, PL = pigmentation loss, CNSN = central nervous system necrosis, TN = tail necrosis, SE = small eyes, SH = small head. Representative figures from a sample of 20 embryos, see additional file for more examples

While non-injected WT embryos did not show any exacerbated morphological defects (embryo controls non-injected or injected with CRISPR dilution buffer did not differ significantly, as assessed in pilot tests), the surviving mutants presented various unusual phenotypes that persisted until later developmental stages. They include abnormal head and brain development without hemorrhage but with apparent necrosis, small eyes with hypopigmentation, body axis with a spinal defect, yolk sac and pericardial edema, swim bladder loss, and tail defects. Most KOs showed several abnormalities (Fig. 5).

### *Natterin-like* gene LOC795232 loss assessed through morphometric analysis.

**Fig. 6:**
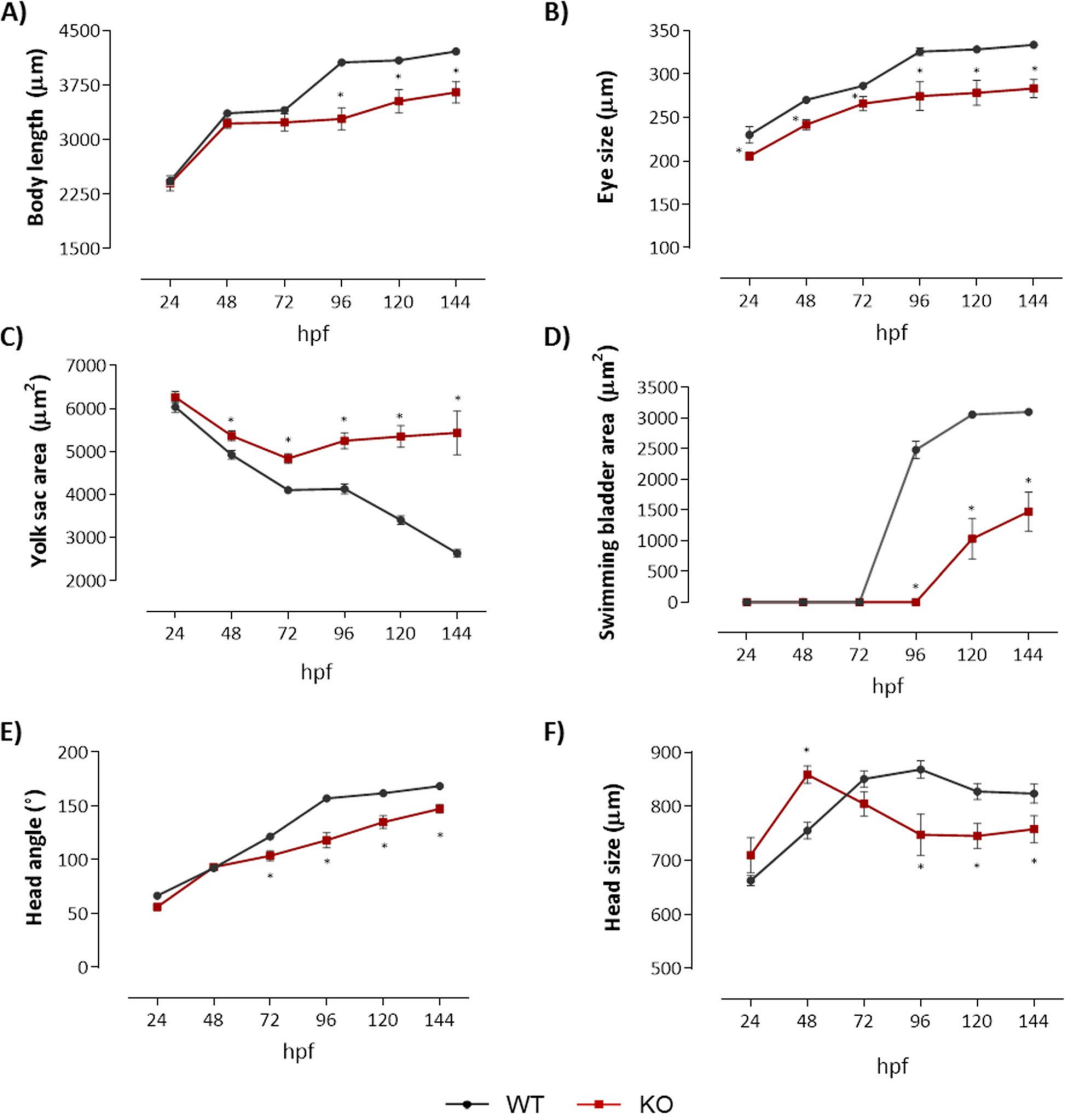
Depletion of natterin-like protein (*LOC795232*) led to kinetic alterations in zebrafish development. The *natterin-like* gene depleted (KO) or the non-depleted larvae (WT) were analyzed at 24, 48, 72, 96, 120, and 144 h post-fertilization (hpf). The larvae were aligned and photographed (20 per group) by Leica M205C stereomicroscope and the measurements of body size (μm, from the top of the head to the end of the tail) (**A**), eye size (μm) (**B**), yolk sac area (μm^2^) (**C**), swimming bladder area (μm^2^) (**D**), head angle (in degrees, given by the measure of the opening of the head in relation to the yolk sac) (**E**), and head size (μm, the antero-caudal measurement of the forebrain to the end of the hindbrain) (**F**) were evaluated in the ImageJ software. The lines represent the average of abnormalities at each designated time. The asterisks (*) represent a significant difference with the WT (*p* < 0.05)

To better understand the involvement of the *natterin-like* in embryonic development, we performed a morphometric analysis in zebrafish mutants and compared to non-injected WT embryos (20 per group; Additional file 3). First, body length was measured as an indication of growth impairment. We observed that in comparison to WT, KOs embryos showed a growth arrest from 96 hpf, resulting in a 13% reduction in the body length at 144 hpf (Fig. 6A). Although we observed that the eyes of the mutants grew over time, they ended up 15% smaller than the control (Fig. 6B; Additional file 2).

In contrast to the continuous growth of the eyes, WT had a progressive decrease in the yolk sac area directly related to its expected absorption. However, the mutants kept larger yolk sacs over time, with an increase of 106% compared to WT, resulting not just due to the low consumption but also because of the edemas recorded (Fig. 6C). As shown in Fig. 4B, all mutants had not shown a swim bladder at 96 hpf, an important element in the larval development, directly involved in posture, fluctuation, and swimming [31]. However, those who developed it showed a 66.3% decrease in the swim bladder area at 120 hpf and 52.5% at 144 hpf compared to WT (Fig. 6D; Additional file 2).

The head-trunk angle, a measurement of the overall head and axial skeleton development, increased primarily between the periods of segmentation and hatching (20 to 70 hpf) as a consequence of the embryo’s straightening [32]. In Fig. 6E, we observed that the *natterin-like* depletion modulated the detachment of the head-trunk, decreasing the angle after 72 hpf compared to WT, with a smaller angle (13%) at the end of the analysis.

In zebrafish, the smaller size of the brain may be the result of reduced neural crest cells and muscles that develop from the paraxial mesoderm [33]. Herein, while the mutants presented similar head sizes to the WT at 24 hpf, at 48 hpf KO heads increased by 14%. However, between 96 and 144 hpf, KO heads were smaller than WT (14, 10, and 8%) (Fig. 6F; Additional files 2 and 3).

### *Natterin-like* gene LOC795232 regulates cardiac and behavioral functions

**Fig. 7:**
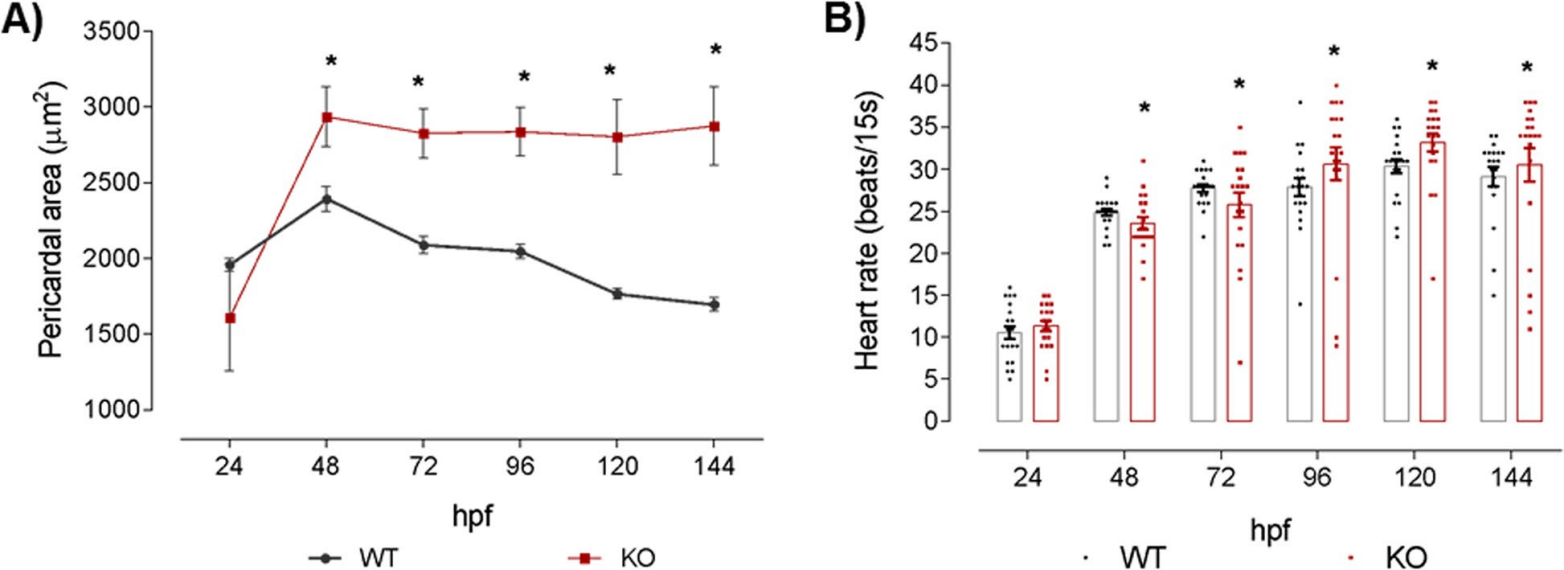
Cardiac alterations of *natterin-like* depleted (KO) zebrafish larvae. The KO or the wild-type (WT) larvae had the heart area and heartbeat rate measured at 24, 48, 72, 96, 120, and 144 h post-fertilization (hpf; 20 larvae per time). Pericardial measurement was evaluated using the ImageJ software from images obtained in a Leica M205C stereomicroscope. The data represent the average of each measurement at the designated time (**A**; 20 larvae per group). The beat rate was counted in 15 s videos acquired on the Leica M205C stereomicroscope at 50x magnification (LAS V4.11 software). Dot plot data show heart rate individually and the bars represent the mean of each group plus the standard deviation (**B**). The asterisks (*) represent a significant difference with the WT control (*p* < 0.05)

The zebrafish linear heart tube begins synchronized contractions around 24 hpf and supports a rudimentary circulatory system with red blood cells (erythrocytes) flowing through the dorsal aorta and cardinal vein, the latter of which only fully connects to the heart later [34]. We analyzed the influence of *natterin-like* on cardiac function by measuring the pericardium area and heart rate. We observed an increase in the pericardial area by 69% at 144 hpf in KO embryos (Fig. 7A). Again, it might be explained by the high incidence of pericardial edema recorded.

Pronounced cardiac malformation coupled with pericardial edema (Fig. 4B) led us to evaluate whether the depletion of *natterin-like* modulated the heartbeat rate. The heartbeat count revealed a continuous increase from 24 to 72 hpf in WT embryos, maintained until 144 hpf (Additional file 2). However, gene depletion induced bradycardia at 48 and 72 hpf in contrast to tachycardia from 96 to 144 hpf (Fig. 7B).

**Fig. 8:**
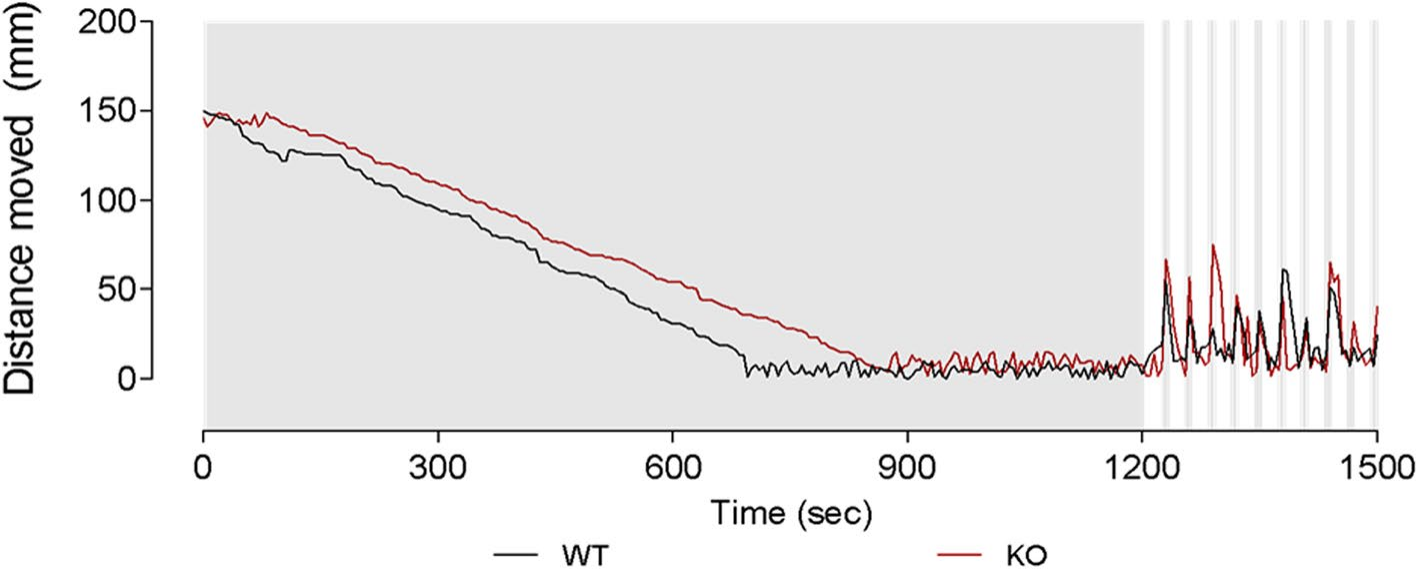
Locomotor analysis of natterin-like depleted (KO) zebrafish larvae. The 144 h post-fertilization (hpf) KO or wild-type (WT) larvae were distributed in a 96-well plate (1 larva/well; 20 larvae per group) in 100 μL of medium and the locomotor activity represented by the distance moved was analyzed through the Zebrabox system (ViewPoint). Larvae were exposed to an acclimatization period of 20 min in the dark followed by 5 min of alternated 25 s light cycles (15% light stimulus) interspersed with 5 s dark cycles (0% light stimulus) to induce visual and neurological stimulation

Since our analysis showed that *natterin-like* depletion promoted changes in several structures directly related to the movement, such as swim bladder, spine, head, and eyes, along with decreased size, our last step was to evaluate the locomotor and behavioral activity. WT larvae had a normal swimming pattern after the dark-light period exposition, as shown in Fig. 8. However, we observed that KO had slightly less activity recorded during the light period, followed by hyperactivity during the dark period (*p* = 0.0140; Fig. 8). Even during the acclimatization period, it is noticeable that the KO larvae were rather agitated and distinguishable from the movement pattern demonstrated by WT animals.

## Discussion

The first members of the Natterin family were described as toxins present in a venomous fish [35]. Afterwards, *natterin-like* were demonstrated in non-venomous fish and other divergent species, where they were assumed to participate in the immune response.

By in silico screening on available genome databases we previously identified 331 species presenting 859 *natterin* or *natterin-like* genes, distributed throughout all kingdoms of life, including plants, fungi, and sessile marine animals with primitive anatomical organization, and teleost fish [36]. Interestingly, although fish represent the majority of species that contain *natterin-like* genes (109 species with 598 sequences), only four species are venomous presenting venom apparatus, i.e., *Plotosus canius*, *Plotosus lineatus*, *Thalassophryne amazonica*, and *T. nattereri* [36, 37].

Members of the family may have roles in development since many of them show distinct importance in embryonic progress as well as in multifunctional fish tissues such as skin, gills, and intestines of the arctic charr (*Salvelinus alpinus)* [38], lumpsucker (*Cyclopterus lumpus)* [39, 40], and ovate pompano (*Trachinotus ovatus)* [41]. Additionally, Cokus et al. [42] identified among numerous genes that *aep1* was highly expressed in the embryonic skin of zebrafish, which was not previously reported to be expressed in the skin during early development.

Employing a combination of the CRISPR/Cas9 depletion system, phenotype-based screening, and morphometric methods, we evaluated the specific function of one family member, the natterin-like protein (XP_017212453.1) encoded by the gene *LOC795232* in comparison to WT non-injected controls. This representative protein is highly similar to *aep1*, which is classified as a sugar-binding natterin-like protein and described for receptor recognition and pore formation. In addition, it is activated early in vertebrate’s development, before the maturation of the adaptive immune system [15].

Even the gene product annotations regarding aep1 point to molecular functions strongly associated with binding mechanisms, and the cellular components expressing this gene are related to the cell periphery, membrane component, and pore complex, reinforcing its designated functions as pore-forming protein, just like all the natterin- and aerolysin-like proteins. Membrane binding may trigger drastic conformational changes of the aerolysin domain in an environmental-dependent manner, resulting in the membrane-bound octameric pore formation. Besides, deeper analysis suggests a process with a distinct activation mechanism from the previously characterized in prokaryotic members [13].

In the CRISPR/Cas9 system, the dgRNA design was achieved by bioinformatics algorithms with a 97.56% predicted specificity and 63.6% efficiency, confirming the recent findings of Naert et al. [43] that indicates a probability above 80% of a projected dgRNA induce frameshift mutations and generate at least 64% efficiency of mutant biallelic cells. Further works also demonstrate that mutagenesis by dgRNA-Cas9 RNPs is highly effective at stimulating double strand breaks (DSB)-repair-induced mutations in the zebrafish, even at target sites that appear resistant to the activity of canonical sgRNA-Cas9 RNPs [23], and greatly heritable, reaching up to 86% [44], > 80% [45], or 75–99% [46], depending on the study.

Following delivery of dgRNPs into zebrafish eggs, roughly all cells present in developing embryos harbor bi-allelic indel mutations [23, 47]. It appears that dgRNPs rarely have off-target effects with phenotypic consequences in the zebrafish [23]. Preeminently, this complex can induce targeted genetic modifications in zebrafish embryos comparable to those obtained using zinc-finger nucleases (ZFNs) and transcription activator-like effector nucleases (TALENs), although presenting the lowest rate of side effects with negligible off-targets. Mutation rates at potential off-target sites are only 1.1–2.5% [44, 47].

The detection of expressed mRNAs through WISH is a robust tool for assessing the spatial distribution of gene transcripts [48]. The zebrafish is especially suitable for this analysis and there are many described gene expression patterns available online at the Zebrafish Information Network (ZFIN, https://zfin.org/action/expression/search/) [47, 49, 50].

The highest expression of *natterin-like* at 24 hpf coincides with the beginning of the differentiation of primitive myeloerythroid lineage within the intermediate cell mass (ICM) [51] and with the emergence of the cardiac contractions and the bloodstream [52]. In zebrafish embryos, primitive hematopoietic cells derived from lateral mesoderm arising in two regions, i.e., the posterior caudal region of the animal between the notochord and endoderm of the trunk, the ICM; and the second in the anterior lateral plate mesoderm (ALPM), a more anterior location under the head. These locations coincide with the higher site-specific expression of the *natterin-like* in the embryos at 24 hpf. The ICM is further divided into the anterior-trunk domain and the posterior blood islands, which form slightly later in the ventral region of the tail. Erythrocytes develop exclusively in the ICM, while the ALPM is the primary site for early macrophage precursors and other myeloid cell types origin [53, 54].

It is noteworthy to point out the persistent expression of *natterin-like* up to at least 144 hpf in the head region, where the zebrafish’s self-renewable hematopoietic stem cells (HSCs) seed the pronephros/primitive kidney, indicating induction of *natterin-like* during hematopoiesis and validating its contribution to this process. HSPCs embedded in the caudal hematopoietic tissue might serve as a source of embryonic macrophages, neutrophils, and monocytes. The kidney, which corresponds to the mammalian bone marrow, will produce myeloid, erythroid, thromboid, and lymphoid lineages, leaving the thymus to produce mature lymphoid T cells throughout adulthood [51].

Zebrafish epiboly demands the synchronized vegetal pole expansion of the blastoderm and yolk syncytial layer. That is used widely during animal development; thus, knowledge acquired by studying epiboly in zebrafish is likely to be relevant for understanding the process in other systems and contexts [55]. Epiboly delay was first evident in KO embryos at 5 hpf after the maternal-to-zygotic transition phase [56]. Then, at the 11 hpf stage, we observed that KO embryos did not present complete blastopore closure at the end of the gastrulation period. Since somites are generated every 30 min in zebrafish [57], the depletion of *natterin-like* led to a decrease in the number of somites in KO embryos during the segmentation period. Defects in the spine and shortening of the body length observed in *natterin-like* deficient larvae may be associated with initial defects in the formation of somites that in turn lead to skeletal and muscle deformities in zebrafish [58].

These results reveal *natterin-like* gene products of zygotic origin playing roles in zebrafish embryo morphogenesis and suggest that their absence may disrupt developmental processes. Most of the major signaling pathways, e.g., TGF-β, FGF, Wnt, Delta-Notch, and retinoic acid, have been identified to drive vertebrate mesoderm development, and many of their interactions have been elucidated. For example, Nodal, BMP, Wnt, and FGF pathways communicate in complex ways to specify both cell fate and cell movement during gastrulation [59]. Wnt, FGF, and Delta-Notch pathways interact with associated transcription factors to direct segmentation [60]. Finally, BMP, Notch, and Wnt pathways interact with associated transcription factors to regulate blood and vessel formation [61].

In addition, the decreased KO eye size may be attributed to the increased activity of Wnt/β-catenin promoted by the absence of *natterin-like* [62]. *Masterblind* (*mbl*)/*axin1* mutant zebrafish that have constitutively hyperactive Wnt signaling lack or have highly reduced forebrain and retina [63]. As described by Ueno et al. [64], Wnt/β-catenin signaling in zebrafish embryos, at different times of development, promotes cardiac differentiation before the gastrulation stage and inhibits it later, which led us to associate the control of this pathway with *natterin-like*. Furthermore, we might link the absence of the bladders in *natterin-like* depleted larvae with modulation of the Hedgehog signaling pathway, which is involved in its development [28].

Furthermore, zebrafish have become increasingly used in behavioral neuroscience. Although the fish brains are smaller and simpler, the genetic, neuronal, and physiological mechanisms that drive behavioral responses to a variety of stimuli are similar to those observed in mammals [65, 66]. The locomotor activity of the 144 hpf zebrafish depends on the integrity of brain function, nervous system development, and visual pathways [67]. Our data showing locomotor hyperactivity in the KO larvae indicate that the absence of the gene may also have affected this complex behavior circuit and, because of that, influence other neurological relevant functions. As a matter of fact, several behavioral responses are the result of cognitive processes, which depend upon structural, physiological, and biochemical characteristics of the central nervous system [65]. Behavioral changes and visual-motor deficiency, for example, after genome editing, have recently been reported by Zhu et al. [68] and Safarian et al. [69], among others.

In this work, it seems that even though the zebrafish have a considerable number of protein copies from the Natterin family, the knockout of one of them caused phenotypic problems during early development. Such abundance in the *natterin-like* representatives recorded in zebrafish might be due to the teleost-specific (Ts3R) whole-genome duplication (WGD) event. The teleost fish underwent a third genome duplication round in addition to those that occurred in the ancient vertebrate lineage [70, 71]. As a consequence of the genomic enlargement and rearrangements during the Ts3R-WGD, the taxon experienced an increase in morphological complexity and innovation, and wide speciation, becoming the most diverse vertebrate group on Earth [72]. However, there are predominantly two ways duplicates may follow, i.e., subfunctionalization, where following genome duplication, the two gene copies degenerate to perform complementary functions that jointly match that of the single ancestral gene; or one copy maintains the ancestral demand while the other diverge, known as neofunctionalization [73]. The last phenomenon explains why each protein may possess a unique role in the development, and its loss results in failures. Besides, regarding the Natterin family, it might explain the broader diversity of functionalities designated to natterin-like proteins, which could be investigated in a molecular evolution study of these genes in more fishes and vertebrates in the future.

Compared to zebrafish, the other species where the four founder natterin members were described, *T. nattereri*, has fewer genes encoding natterin proteins. This discrepancy is probably because about 75% of the genes from the Ts3R-WGD event may revert to singletons [74]. On the other hand, the duplication-degeneration-complementation (DDC) hypothesis [75, 76] has been proposed as an explanation for the high retention of duplicate genes, which also suggests that genes with simple tissue- and time-specific regulatory elements would be more likely to revert to singletons than those with complex regulation. Thus, we infer that *LOC795232* can be both involved in multifunctional processes and share roles with other natterin-like proteins, such as *aep1*.

Throughout evolution, mainly immune molecular families have expanded in some species, providing critical functional effects against pathogens. Proteins of the Natterin family may be evidence of this diversification. Even the Agnathans, a superclass of jawless fish and the most primitive members of the vertebrates, present a natterin family member, which implements variable lymphocyte receptors (VLRs)-activated complement cytotoxicity for antigen recognition [10]. Moreover, a natterin-like protein found in zebrafish (*aep1*) with high homology with the one from the lamprey was identified to be a putative immune defense molecule by forming pore-like structures like aerolysin [13]. Ultimately, Chen et al. [15] suggested that *aep1* may be a pro-inflammatory protein and an innate immune molecule that triggers the antimicrobial immune responses preventing zebrafish from bacterial infection. Overall, considering the concepts discussed before, to different extents, the *natterin-like* genes are potentially primitive and important agents in development and immune response.

## Conclusions

The present work provided the first demonstration of the *natterin-like* role in embryonic development using zebrafish. Depletion generated mutants with abnormal phenotypes that worsened over time and died prematurely. The severe KO phenotypic abnormalities included curved body axis with small bodies, head and eyes deformities, with lack or reduced swim bladders, frequently accompanied by pericardial and yolk sac edema. These abnormalities affected the zebrafish’s physiologically relevant functions, leading to severe heart dysfunction and a locomotor hyperactivity pattern suggestive of high levels of stress/anxiety.

Taken together, these results demonstrated that programmable CRISPR/Cas9 systems provide a precise, rapid, and reliable method for altering genes in vivo*.* It opens the venue to using RNA-guided nucleases for genome editing remarkably in the zebrafish, which is highly validated as a powerful vertebrate model for investigating gene functions and translational research on developmental disorders, thereby demonstrating its potential as a simple, customizable and ready-to-use genome editing tool. The emergence of zebrafish genome editing offers promising new opportunities to understand the genetic basis of *natterin-like* roles employing targeted mutations. The study of zebrafish through morphogenesis and embryogenic development can provide compelling and broadly applicable insights into the genetic, molecular, and cellular control of the Natterin family members. The knowledge over this family is essential for a better comprehension of these proteins since they are not just overspread around fish but also in organisms from a diversity of taxons and there is increasingly growing evidence of their fundamental contributions to the development and immune response.

## Methods

### Multiple sequence alignment

For the multiple alignments and selection of the gene, we used all known zebrafish natterin-like protein sequences obtained from the National Center for Biotechnology Information (NCBI) aligned through the multiple sequence alignment tool from the software Clustal Omega [European Molecular Biology Laboratory - The European Bioinformatics Institute (EMBL-EBI); https://www.ebi.ac.uk/Tools/msa/clustalo/]. The alignment was visualized in the viewer MView (https://www.ebi.ac.uk/Tools/msa/mview/). Clustal aligns sequences using a heuristic method that progressively builds a multiple sequence alignment from a series of pairwise alignments. Essentially, Clustal creates multiple sequence alignments through three main steps: performing a pairwise alignment using the progressive alignment method, creating a guide tree, and using the guide tree to carry out multiple alignments.

### Zebrafish husbandry

Adult zebrafish (< 18 months old) from AB strain (International Zebrafish Resource Center, Eugene, OR, US) were kept separated by sex and bred under standard conditions of temperature at 28 °C, pH 7, and light-dark cycle (14/10 h) in individual aquariums in an ALESCO (Campinas, Brazil) rack using system water (60 μg.mL^− 1^ Instant Ocean sea salts). The experiments were carried out under the laws of the National Council for Animal Experiment Control (CONCEA) and approved by the Butantan Institute’s Ethics Committee on the Use of Animals (CEUAIB #6.888.280.519 and #2.648.280.519). The study was performed and is reported in accordance with ARRIVE guidelines. The fertilized embryos checked in the Leica EZ4W stereomicroscope (Leica Microsystems, Cambridge, UK) were transferred to 100 × 25 mm plastic dishes (#89107–632, VWR) containing 0.5x E2 medium (7.5 mM KH_2_PO_4_, 2.5 mM Na_2_HPO_4_, 15 mM NaCl, 0.5 mM KCl, 1 mM MgSO_4_.7H_2_O, 1 mM CaCl_2_.2H_2_O, 0.7 mM NaHCO_3_) and classified according to Kimmel et al. [32].

### Zebrafish anesthesia, dechorionation, and euthanasia

Anesthesia was performed by immersing larvae in 2 mL of 0.5x E2 medium containing 0.4% tricaine (ethyl-3-aminobenzoate, #MS-222, Sigma Chemical Co., St. Louis, MO, US) for 2 min at room temperature before analysis. At the end of the experiments, euthanasia was obtained by immersion in 4% tricaine diluted in 0.5x E2 medium. After exposure, larvae were checked in an M205C stereomicroscope (Leica Microsystems) to ensure that their hearts were not beating before being placed in a 10% bleach solution. When suitable, 24 hpf larvae were anesthetized and dechorionated by immersion in pronase (#P5147, Sigma) at 0.02 mg.mL^− 1^ for 5 min.

### *Danio rerio Natterin-like* Gene CRISPR/Cas9 System

The *natterin-like* gene *LOC795232* (https://www.ncbi.nlm.nih.gov/gene/795232, XM_017356964, chromosome 7, 966 bp) was used for the CRISPR/Cas9 system construction, which was composed by the duplex guide RNA (dgRNA)-Cas9 ribonucleoprotein (RNP) complex [tracrRNA (TRACRRNA05N) + natterin-like crRNA (5′-3′: CAGAATGTCAAATAGATGT - WD0747994), Sigma-Aldrich] with Cas9 protein [CAS9PROT contains RuvC/ribonuclease (RNase) H domain, Sigma-Aldrich] resuspended in nuclease-free DEPC water (#750024, Invitrogen, Life Technologies Van Allen Way Carlsbad, CA, US). Different combinations of dgRNA:Cas9 (concentrations in μg.nL^− 1^) were previously tested to standardize the combination that provided an efficient depletion of the target (*natterin-like*) without pronounced side effects related to the gene-editing system. They were diluted in 1× Danieau buffer (58 mM NaCl, 0.7 mM KCl, 0.4 mM MgSO_4_, 0.6 mM Ca(NO_3_)_2_, 5.0 mM HEPES; pH 7.6) containing 1% phenol red (#P3532, Sigma). The depleted larvae (KO) were compared to the non-injected control group (WT), since preliminary data confirm that non-injected embryos and those injected with dgRNA-Cas9 dilution buffer (DBUFFER, Sigma-Aldrich) did not differ significantly in terms of survival and phenotypic alterations (data not shown). Cas9 RNPs complexes consisting of synthetic crRNA:tracrRNA duplex that perfectly matches the target site are highly mutagenic in zebrafish embryos and effective at inducing mutations in F0 zebrafish. Mutagenized F0 embryos mimic null mutants and lack confounding non-specific traits [23].

### Microinjection in zebrafish

Anesthetized 0 hpf or one-cell stage embryos, mounted into an agarose-coated plate groove (#16500100, Invitrogen, Carlsbad, California, EU) were injected using M205C stereomicroscope (Leica Microsystems) using a microneedle (#5242952008 femtotips 930,000,043, 0.5–0.7 μm Eppendorf, Hamburg, DE) coupled to the Injectman® 4 pneumatic micromanipulator microinjector (Eppendorf, Hamburg, DE) pressurized with approximately 2–3 nL of CRISPR/Cas9 system into the cell, previously calibrated using micrometer-scale 1 mm in 0.01 mm divisions slide (#2280–13-1EA, Ted Pella). After injection, embryos were incubated in 0.5x E2 medium at 28 °C and analyzed after 24, 48, 72, 96, 120, and 144 hpf.

### Evaluation of embryonic survival and development

Mortality (egg coagulation) was checked daily as well as the occurrence of deformities in the surviving embryos, such as the absence of somites and non-detachment of the tail (lethality parameters); head/eye malformation, abnormal yolk sac absorption, pericardium and yolk sac edema, uninflated or absent swimming bladder, and abnormal pigmentation (sublethality parameters); curved tail or shortened tail, spinal deformity, and delayed growth (teratogenicity parameters) were analyzed according to Shah et al. [77]. The images obtained using an M205C stereomicroscope (Leica Microsystems) at 27x magnification were used for measurements using the ImageJ v.1.8.0_172.

### Evaluation of zebrafish epiboly

Whole embryo images were collected from non-dechorionated animals aligned in a 1.2% agarose support and covered by 0.5x E2 medium. Images were acquired (27x magnification) every 1 h until 24 h of development in stereomicroscope Leica M205C (LAS V4.11 software) for analysis of epiboly initiation at 5 hpf when the yolk cell domes and deep cells move radially outwards, forming a cap of cells over the yolk; during the progression phase (11 hpf) when the blastoderm continues to thin, expanding its surface area to envelop the yolk cell; and at 19 hpf when the blastoderm has covered approximately 50% of the yolk when deep cell epiboly temporarily pauses as cells begin to converge dorsally until gastrulation begins [55].

### *Whole-Mount* in situ Hybridization (WISH) for Detection of the *Natterin-like* Gene

The 72 hpf embryos microinjected with CRISPR/Cas9 (KO) or non-injected controls (WT) were fixed in fresh 4% formaldehyde overnight at 4 °C. Then, the embryos were dehydrated in methanol 100% and stored at − 80 °C. Embryos were removed from the ultrafreezer and rehydrated with gradient dilutions of methanol in PBS (75, 50, and 25%). The embryos were digested with 10 μg.mL^− 1^ of proteinase K (#P6556, Sigma-Aldrich) in 200 μL of proteinase K buffer (0.005 M Tris-HCl, 0.001 M EDTA, 0.001 M NaCl in RNase-free water) at room temperature for 30 min and then rinsed in formaldehyde 4% for 20 min to stop the digestion, followed by four rounds wash with PBST [1x PBS, 0.1% Tween 20 (v/v)] to remove the formaldehyde residues. Later, they were incubated in 200 μL of complete hybridization buffer (50% formamide in 5x SSC buffer, 0.1% tween 20, 500 μg.mL^− 1^ yeast RNAse (#AM9789, Ambiam) and 50 μg.mL^− 1^ heparin (#CC-4396A, Lonza), pH 6) for 3 h at 70 °C. After, samples were incubated overnight with 40 nM of the DIG-labeled *natterin-like* gene detection probe (#339500 LCD0168623 BKG LOC795232_1, miRCURY LNA miRNA, Qiagen) at 70 °C. In the next day, the probe was removed by washing with incomplete (without heparin or RNAse) hybridization buffer in 2x SSC (75, 50, and 25%) and incomplete hybridization buffer in 0.2x SSC (75, 50, and 25%) for 10 min each at room temperature. Then, the embryos’ non-specific antibody sites were blocked with blocking solution (1x PBST, 2% tilapia serum, and 2 mg.mL^− 1^ BSA) for 3.5 h at room temperature. Anti-DIG-AP (#11093274910, Roche Diagnostics) at 1:300 dilution in blocking solution was added and agitated (40 rpm) overnight at 4 °C. The embryos were washed with PBST six times for 15 min at room temperature. Then, the embryos were soaked in 200 μL of fresh staining solution prepared with 50 mg.mL^− 1^ BCIP (5-Bromo-4-chloro-3-Indolyl phosphatase; #11383221001, Roche Diagnostics) and 100 mg.mL^− 1^ NBT (4-nitro blue tetrazolium chloride; #11383213001, Roche Diagnostics) in the dark at room temperature for 4 h, monitored in a stereomicroscope every 1 h. The colorimetric reaction was stopped by washing the embryos three times in the stop solution (1x PBS, 1 mM EDTA, and 0.1% tween 20, pH 5.5) and fixed with 200 μL of 100% glycerol overnight under agitation (40 rpm) at room temperature. Embryos were visualized on AxioVision® software (Carl Zeiss, Oberkochen, DE) in 60 and 100x magnification. The qualitative expression of the *natterin-like* gene was confirmed in the entire larva by an intense blue-purple precipitate signal [21].

### Phenotype-based screening

The WT and KO embryos were anesthetized, aligned in a glass dish in the lateral position, and photographed under an M205C stereomicroscope (Leica Microsystems). The images obtained were used according to the methods previously reported by Bilder et al. [29]. For measurement of the body length, they were evaluated horizontally from the top of the head to the tip of the tail (μm); the head size evaluated by the antero-caudal measurement of the forebrain, midbrain, and hindbrain (μm); the yolk circumference area (μm^2^); the eye diameter (μm); the swim bladder circumference area (μm^2^); the angle of the head (in degree, °) evaluated by the opening of the head in relation to the yolk sac; and the pericardial area (μm^2^) using ImageJ v.1.8.0_172.

### Cardiac function analysis

The anesthetized zebrafish embryos of the WT or KO groups, aligned side by side in groups of 5, were recorded for 15 s per day for 6 days in M205C stereomicroscope (Leica Microsystems; LAS V4.11 software) at 80x magnification for the heart rate evaluation by heartbeats count.

### Zebrafish locomotor behavior assessment

The evaluation of locomotor activity was carried out by analyzing the swimming behavior of 144 hpf zebrafish larvae upon the dark-light transition according to the modified method of Macaulay et al. [78]. WT and KO larvae (*n* = 20) were transferred to 96-well plates, one larva per well in 100 μL of 0.5x E2 medium, and analyzed in a Zebrabox System (Viewpoint Life sciences, Lyon, FR). The larvae were analyzed for a total of 25 min; consisting of 20 min of acclimatization in the dark followed by 5 min of 10 cycles of 25 s in the light to induce visual and neurological stimulation followed by 5 s in the dark. Locomotor activity was quantified and analyzed by ZebraLab™ software by Viewpoint.

### Statistical analysis

All values were expressed as mean ± SEM. Experiments were performed independently two times. Parametric data were evaluated using analysis of variance, followed by the Bonferroni correction for multiple comparisons. Non-parametric data were assessed using the Mann-Whitney test. Differences were considered statistically significant at *p* < 0.05 using GraphPad Prism (Graph Pad Software, v6.02, 2013, La Jolla, CA, US).

## Supplementary Information

**Additional File 1. **Zebrafish epiboly. Time-lapse video in brightfield microscopy of a wild-type (WT) (left) and *natterin-like* mutant (KO) (right) embryo. Imaging was performed with 2.1 min time frames at an ambient temperature of 26 °C, lateral view; animal pole is on top, between scale bar: 65 μm.

**Additional File 2. **Embryo measurements. Raw measurement files of wild-type (WT) and *natterin-like* knockout (KO) embryos assessed in phenotype-based and locomotor behavior screening.

**Additional File 3.** Images of embryos from all stages, views, and genotypes. The images were obtained using an M205C stereomicroscope (Leica Microsystems) at 10x magnification with embryos anesthetized with 0.4% tricaine. (PPTX 20745 kb)

## Data Availability

The datasets analyzed during the current study are available in the National Center for Biotechnology Information repository, https://www.ncbi.nlm.nih.gov/gene/?term=(natterin) + AND + %22Danio + rerio%22%5Bporgn3A__txid79555D, at NCBI gene search: “(natterin) AND “*Danio rerio*”[porgn:__txid7955]”.

## Abbreviations

V*Tn*: *Thalassophryne nattereri* venom
CRISPR: Clustered regularly interspaced short palindromic repeats
dgRNP: duplex guide ribonucleoprotein
aa: Amino acids
PID: Percentage of identity
WT: Wild-type
KO: Null mutant
hpf: Hours post-fertilization
tracrRNA: Trans-activating crRNA
WISH: Whole-mount in situ hybridization
dgRNA: Duplex guide RNA
DSB: Double strand breaks
ZFN: Zinc-finger nucleases
TALEN: Transcription activator-like effector nuclease
dpf: days post-fertilization
ICM: Intermediate cell mass
HSC: Hematopoietic stem cell
Ts3R-WGD: teleost-specific whole-genome duplication
VLR: Variable lymphocyte receptors
NCBI: National Center for Biotechnology Information
HMM: Hidden Markov model
CONCEA: National Council for Animal Experiment Control
CEUAIB: Butantan Institute’s Ethics Committee on the Use of Animals
hpf: Hours post-fertilization

## References

[1] Magalhães GS, Junqueira-de-Azevedo ILM, Lopes-Ferreira M, Lorenzini DM, Ho PL, Moura-da-Silva AM (2006). Transcriptome analysis of expressed sequence tags from the venom glands of the fish *Thalassophryne nattereri*. Biochimie..

[2] Fonseca LA, Lopes-Ferreira M (2000). Clinical and experimental studies regarding poisoning caused by a fish *Thalassophryne nattereri* (niquim). Anais Bras Dermat.

[3] Komegae EN, Ramos AD, Oliveira AK, Toledo Serrano SM, Lopes-Ferreira M, Lima C (2011). Insights into the local pathogenesis induced by fish toxins: role of natterins and nattectin in the disruption of cell-cell and cell-extracellular matrix interactions and modulation of cell migration. Toxicon..

[4] Komegae EN, Grund LZ, Lopes-Ferreira M, Lima C (2013). The longevity of th2 humoral response induced by proteases natterins requires the participation of long-lasting innate-like B cells and plasma cells in spleen. PLoS One.

[5] Komegae EN, Grund LZ, Lopes-Ferreira M, Lima C (2013). TLR2, TLR4 and the MyD88 signaling are crucial for the in vivo generation and the longevity of long-lived antibody-secreting cells. PLoS One.

[6] Bruni FM, Coutinho EMM, Andrade-Barros AI, Grund LZ, Lopes-Ferreira M, Lima C (2020). Anaphylaxis induced by *Thalassophryne nattereri* venom in mice is an IgE/IgG1-mediated, IL-4-dependent phenomenon. Sci Report Sci Rep.

[7] Lima C, Falcao MAP, Andrade-Barros AI, Seni-Silva AC, Grund LZ, Balogh E (2021). Natterin an aerolysin-like fish toxin drives IL-1β-dependent neutrophilic inflammation mediated by caspase-1 and caspase-11 activated by the inflammasome sensor NLRP6. Int Immunopharmacol.

[8] Holm HJ, Wadsworth S, Bjelland AK, Krasnov A, Evensen Ø, Skugor S (2016). Dietary phytochemicals modulate skin gene expression profiles and result in reduced lice counts after experimental infection in Atlantic salmon. Parasit Vectors.

[9] Xue Z, Liu X, Pang Y, Yu T, Xiao R, Jin M (2012). Characterization, phylogenetic analysis and cDNA cloning of natterin-like gene from the blood of lamprey, *Lampetra japonica*. Immunol Lett.

[10] Wu F, Feng B, Ren Y, Wu D, Chen Y, Huang S (2017). A pore-forming protein implements VLR-activated complement cytotoxicity in lamprey. Cell Discovery.

[11] Neave MJ, Sunarto A, McColl K (2017). Transcriptomic analysis of common carp anterior kidney during cyprinid herpesvirus 3 infection: immunoglobulin repertoire and homologue functional divergence. Sci Rep.

[12] Rajan B, Patel DM, Kitani Y, Viswanath K, Brinchmann MF (2017). Novel mannose binding natterin-like protein in the skin mucus of Atlantic cod (*Gadus morhua*). Fish Shellfish Immunol.

[13] Jia N, Liu N, Cheng W, Jiang YL, Sun H, Chen LL (2016). Structural basis for receptor recognition and pore formation of a zebrafish aerolysin-like protein. EMBO Rep.

[14] Leprêtre M, Almunia C, Armengaud J, Le Guernic A, Salvador A, Geffard A (2020). Identification of immune-related proteins of *Dreissena polymorpha* hemocytes and plasma involved in host-microbe interactions by differential proteomics. Sci Rep.

[15] Chen LL, Xie J, Cao DD, Jia N, Li YJ, Sun H (2018). The pore-forming protein Aep1 is an innate immune molecule that prevents zebrafish from bacterial infection. Dev Comp Immunol.

[16] Blackburn PR, Campbell JM, Clark KJ, Ekker SC (2013). The CRISPR system—keeping Zebrafish gene targeting fresh. Zebrafish..

[17] Semple F, Dorin JR (2012). β-Defensins: multifunctional modulators of infection, inflammation and more?. J Innate Immun.

[18] Pai P, Sukumar S (2020). HOX genes and the NF-κB pathway: a convergence of developmental biology, inflammation and cancer biology. Biochim Biophys Acta Rev Cancer.

[19] Howe K, Clark MD, Torroja CF, Torrance J, Berthelot C, Muffato M (2013). The zebrafish reference genome sequence and its relationship to the human genome. Nature..

[20] Wang XW, Wang JX (2013). Diversity and multiple functions of lectins in shrimp immunity. Dev Comp Immunol.

[21] Thisse C, Thisse B (2008). High-resolution in situ hybridization to whole-mount zebrafish embryos. Nat Protoc.

[22] Bae S, Park J, Kim JS (2014). Cas-OFFinder: a fast and versatile algorithm that searches for potential off-target sites of Cas9 RNA-guided endonucleases. Bioinformatics..

[23] Hoshijima K, Jurynec MJ, Klatt Shaw D, Jacobi AM, Behlke MA, Grunwald DJ (2019). Highly efficient CRISPR-Cas9-based methods for generating deletion mutations and F0 embryos that lack gene function in Zebrafish. Dev Cell.

[24] Jacobi AM, Rettig GR, Turk R, Collingwood MA, Zeiner SA, Quadros RM (2017). Simplified CRISPR tools for efficient genome editing and streamlined protocols for their delivery into mammalian cells and mouse zygotes. Methods..

[25] Shankaran SS, Dahlem TJ, Bisgrove BW, Yost HJ, Tristani-Firouzi M (2017). CRISPR/Cas9-directed gene editing for the generation of loss-of-function mutants in high-throughput Zebrafish F0 screens. Curr Protoc Mol Biol.

[26] Bruce AEE, Heisenberg CP (2020). Mechanisms of zebrafish epiboly: a current view. Curr Top Dev Biol.

[27] Quinlivan VH, Farber SA (2017). Lipid uptake, metabolism, and transport in the larval Zebrafish. Front Endocrinol.

[28] Winata CL, Korzh S, Kondrychyn I, Zheng W, Korzh V, Gong Z (2009). Development of zebrafish swim bladder: the requirement of hedgehog signaling in specification and organization of the three tissue layers. Dev Biol.

[29] Bilder RM, Sabb FW, Cannon TD, London ED, Jentsch JD, Parker DS (2009). Phenomics: the systematic study of phenotypes on a genome-wide scale. Neuroscience..

[30] Strahle U, Grabher C (2010). The zebrafish embryo as a model for assessing off-target drug effects. Dis Model Mech.

[31] Parichy DM, Elizondo MR, Mills MG, Gordon TN, Engeszer RE (2009). Normal table of postembryonic zebrafish development: staging by externally visible anatomy of the living fish. Dev Dyn.

[32] Kimmel CB, Ballard WW, Kimmel SR, Ullmann B, Schilling TF (1995). Stages of embryonic development of the zebrafish. Dev Dyn.

[33] Ando H, Sato T, Ito T, Yamamoto J, Sakamoto S, Nitta N (2019). Cereblon control of Zebrafish brain size by regulation of neural stem cell proliferation. iScience..

[34] Kemmler CL, Riemslagh FW, Moran HR, Mosimann C (2021). From stripes to a beating heart: early cardiac development in Zebrafish. J Cardiovasc Dev Dis.

[35] Magalhães GS, Lopes-Ferreira M, Junqueira-De-Azevedo ILM, Spencer PJ, Araújo MS, Portaro FCV (2005). Natterins, a new class of proteins with kininogenase activity characterized from *Thalassophryne nattereri* fish venom. Biochimie..

[36] Lima C, Disner GR, Falcão MAP, Seni-Silva AC, Maleski ALA, Marcolino-Souza M (2021). The Natterin protein family diversity: a review on phylogeny, structure, and immune function. Toxins..

[37] Tamura S, Yamakawa M, Shiomi K (2011). Purification, characterization and cDNA cloning of two natterin-like toxins from the skin secretion of oriental catfish Plotosus lineatus. Toxicon..

[38] Gudbrandsson J, Ahi EP, Franzdottir SR, Kapralova KH, Kristjansson BK, Steinhaeuser SS (2015). The developmental transcriptome of contrasting Arctic charr (*Salvelinus alpinus*). Morphs*.* F1000. Research..

[39] Patel DM, Brinchmann MF (2017). Skin mucus proteins of lumpsucker (*Cyclopterus lumpus*). Biochem Biophys Rep.

[40] Patel DM, Bhide K, Bhide M, Iversen MH, Brinchmann MF (2019). Proteomic and structural differences in lumpfish skin among the dorsal, caudal and ventral regions. Sci Rep.

[41] Lin KT, Wang WX, Ruan HT, Dai JG, Sun JJ, Liu L (2019). Transcriptome analysis of differentially expressed genes in the fore- and hind-intestine of ovate pompano *Trachinotus ovatus*. Aquaculture..

[42] Cokus SJ, De La Torre M, Medina EF, Rasmussen JP, Ramirez-Gutierrez J, Sagasti A (2019). Tissue-specific Transcriptomes reveal gene expression trajectories in two maturing skin epithelial layers in Zebrafish embryos. G3 (Bethesda, Md).

[43] Naert T, Tulkens D, Edwards NA, Carron M, Shaidani NI, Wlizla M (2020). Maximizing CRISPR/Cas9 phenotype penetrance applying predictive modeling of editing outcomes in *Xenopus* and zebrafish embryos. Sci Rep.

[44] Hruscha A, Krawitz P, Rechenberg A, Heinrich V, Hecht J, Haass C (2013). Efficient CRISPR/Cas9 genome editing with low off-target effects in zebrafish. Development..

[45] Hwang WY, Fu Y, Reyon D, Maeder ML, Tsai SQ, Sander JD (2013). Efficient genome editing in zebrafish using a CRISPR-Cas system. Nat Biotech.

[46] Irion U, Krauss J, Nüsslein-Volhard C (2014). Precise and efficient genome editing in zebrafish using the CRISPR/Cas9 system. Development..

[47] Burger A, Lindsay H, Felker A, Hess C, Anders C, Chiavacci E (2016). Maximizing mutagenesis with solubilized CRISPR-Cas9 ribonucleoprotein complexes. Development..

[48] Vauti F, Stegemann LA, Vögele V, Köster RW (2020). All-age whole mount in situ hybridization to reveal larval and juvenile expression patterns in zebrafish. PLoS One.

[49] Meneghetti G, Skobo T, Chrisam M, Facchinello N, Fontana CM, Bellesso S (2019). The epg5 knockout zebrafish line: a model to study Vici syndrome. Autophagy..

[50] Sun X, Zhang R, Chen H, Du X, Chen S, Huang J (2020). Fgfr3 mutation disrupts chondrogenesis and bone ossification in zebrafish model mimicking CATSHL syndrome partially via enhanced Wnt/β-catenin signaling. Theranostics..

[51] Bertrand JY, Kim AD, Violette EP, Stachura DL, Cisson JL, Traver D (2007). Definitive hematopoiesis initiates through a committed erythromyeloid progenitor in the zebrafish embryo. Development..

[52] Miura GI, Yelon D (2011). A guide to analysis of cardiac phenotypes in the Zebrafish embryo. Methods Cell Biol.

[53] Berman J, Kanki J, Look A (2005). Zebrafish as a model for myelopoiesis during embryogenesis. Exp Hematol.

[54] Brownlie A, Hersey C, Oates AC, Paw BH, Falick AM, Witkowska HE (2003). Characterization of embryonic globin genes of the zebrafish. Dev Biol.

[55] Bruce AEE (2015). Zebrafish epiboly: spreading thin over the yolk. Dev Dyn.

[56] Schulz KN, Harrison MM (2019). Mechanisms regulating zygotic genome activation. Nat Rev Genet.

[57] Saga Y, Takeda H (2001). The making of the somite: molecular events in vertebrate segmentation. Nat Rev Genet.

[58] McCollum CW, Ducharme NA, Bondesson M, Gustafsson JA (2011). Developmental toxicity screening in zebrafish. Birth defects res. Part C Embryo Today Rev.

[59] Heisenberg CP, Solnica-Krezel L (2008). Back and forth between cell fate specification and movement during vertebrate gastrulation. Curr Opin Genet Dev.

[60] Dunty WC, Biris KK, Chalamalasetty RB, Taketo MM, Lewandoski M, Yamaguchi TP (2008). Wnt3a/B-catenin signaling controls posterior body development by coordinating mesoderm formation and segmentation. Development..

[61] Lee D, Park C, Lee H, Lugus JJ, Kim SH, Arentson E (2008). ER71 acts downstream of BMP, notch, and Wnt signaling in blood and vessel progenitor specification. Cell Stem Cell.

[62] Glass AS, Dahm R (2004). The Zebrafish as a model organism for eye development. Ophthalmic Res.

[63] Van de Wetering M, Joore J, Esseling J, Bink R, Clevers H, Van de Water S (2001). Ectopic Wnt signal determines the eyeless phenotype of zebrafish masterblind mutant. Development..

[64] Ueno S, Weidinger G, Osugi T, Kohn AD, Golob JL, Pabon L (2007). Biphasic role for Wnt/beta-catenin signaling in cardiac specification in zebrafish and embryonic stem cells. Proc Natl Acad Sci.

[65] Luchiari AC, Málaga-Trillo E, Tran S, Gerlai R (2021). Editorial: Zebrafish cognition and behavior. Front Behav Neurosci.

[66] Gerlai R (2020). Evolutionary conservation, translational relevance and cognitive function: the future of zebrafish in behavioral neuroscience. Neurosci Biobeh Rev.

[67] Basnet RM, Zizioli D, Taweedet S, Finazzi D, Memo M (2019). Zebrafish larvae as a behavioral model in neuropharmacology. Biomedicines..

[68] Zhu J, Xu H, Song H, Li X, Wang N, Zhao J (2021). CRISPR/Cas9-mediated grna gene knockout leads to neurodevelopmental defects and motor behavior changes in zebrafish. J Neurochem.

[69] Safarian N, Whyte-Fagundes P, Zoidl C, Grigull J, Zoidl G (2020). Visuomotor deficiency in panx1a knockout zebrafish is linked to dopaminergic signaling. Sci Rep.

[70] Glasauer SMK, Neuhauss SCF (2014). Whole-genome duplication in teleost fishes and its evolutionary consequences. Mol Genet Genomics.

[71] Clarke JT, Lloyd GT, Friedman M (2016). Little evidence for enhanced phenotypic evolution in early teleosts relative to their living fossil sister group. PNAS..

[72] Sato Y, Nishida M (2010). Teleost fish with specific genome duplication as unique models of vertebrate evolution. Environ Biol Fish.

[73] Sandve SR, Rohlfs RV, Hvidsten TR (2018). Subfunctionalization versus neofunctionalization after whole-genome duplication. Nat Genet.

[74] Postlethwait JH, Woods IG, Ngo-Hazelett P, Yan YL, Kelly PD, Chu F (2000). Zebrafish comparative genomics and the origins of vertebrate chromosomes. Genome Res.

[75] Force A, Lynch M, Pickett FB, Amores A, Yan YL, Postlethwait J (1999). Preservation of duplicate genes by complementary, degenerative mutations. Genetics..

[76] MacCarthy T, Bergman A (2007). The limits of subfunctionalization. BMC Evol Biol.

[77] Shah SM, Wahba M, Yu L, Achari G, Habibi HR (2019). Health impact assessment of Sulfolane on embryonic development of Zebrafish (*Danio rerio*). Toxics..

[78] Macaulay LJ, Bailey JM, Levin ED, Stapleton HM (2015). Persisting effects of a pbde metabolite, 6-oh-bde-47, on larval and juvenile zebrafish swimming behavior. Neurotoxicol Teratol.

