# Supplementary figures and images for: Natterin-like depletion by CRISPR/Cas9 impairs zebrafish (Danio rerio) embryonic development
Source: BMC Genomics. 2022 Feb 12;23:123. doi: 10.1186/s12864-022-08369-z (PMC8840632; doi:10.1186/s12864-022-08369-z)

## Slide 1
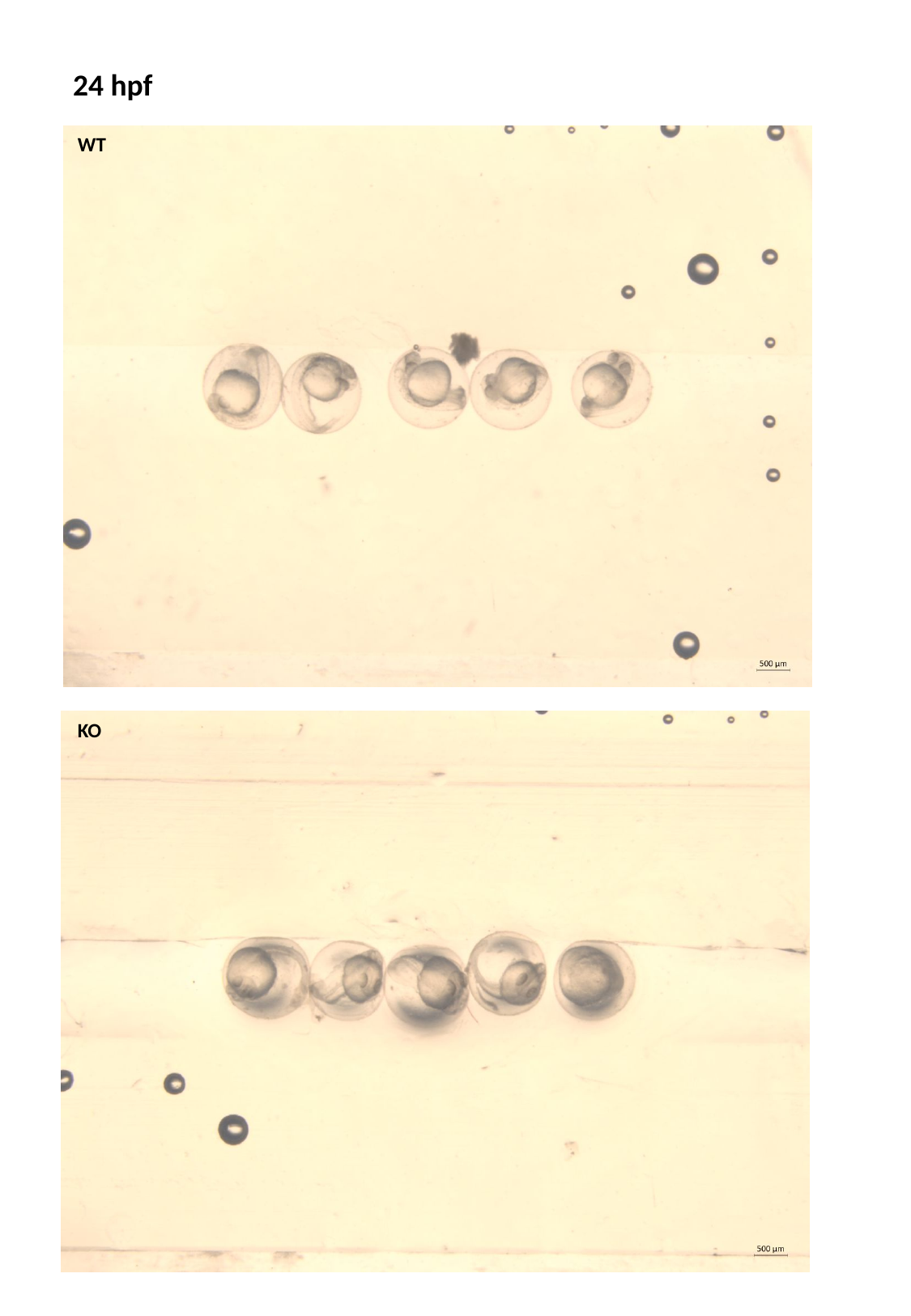

24 hpf
WT
KO

## Slide 2
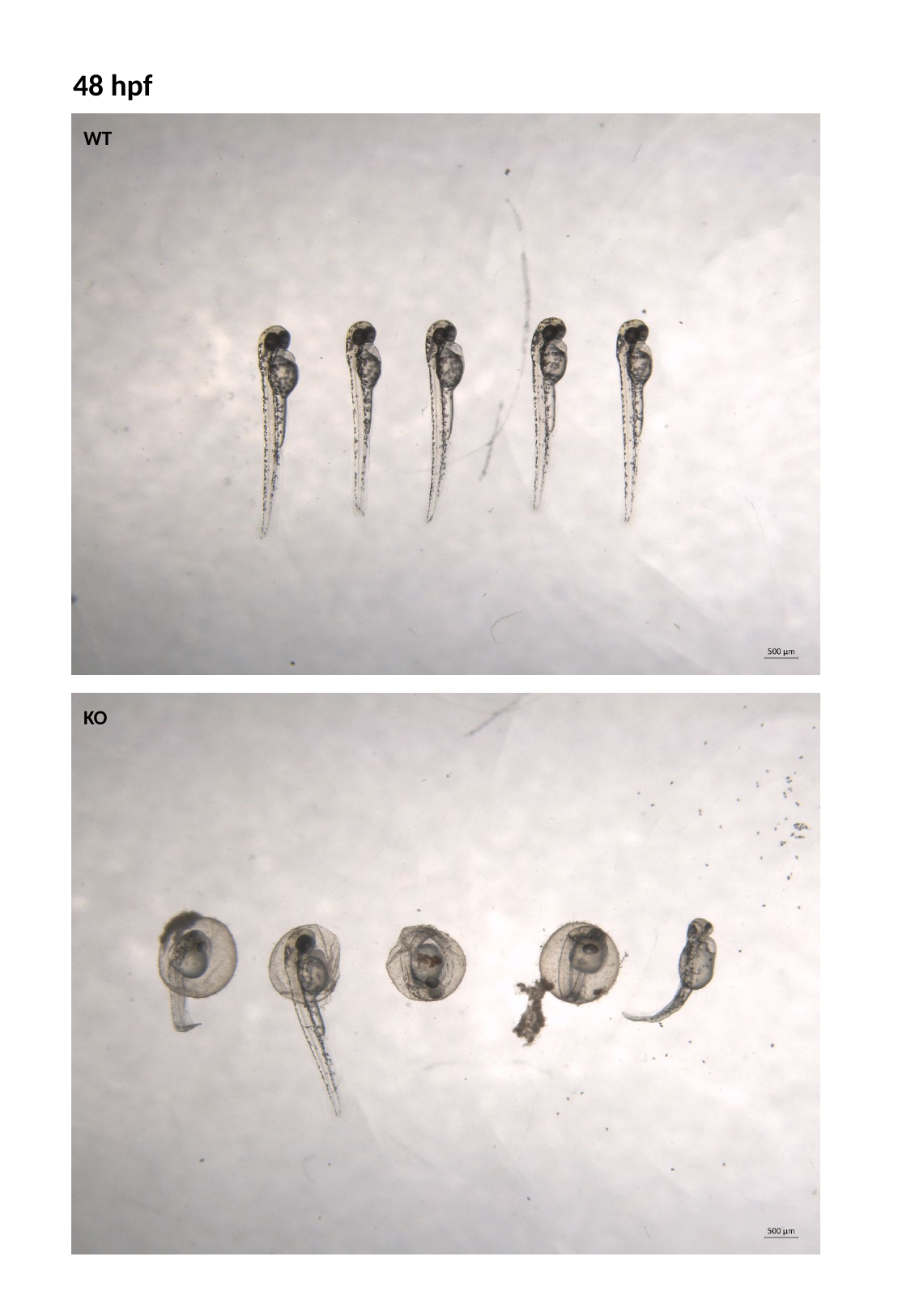

48 hpf
WT
KO

## Slide 3
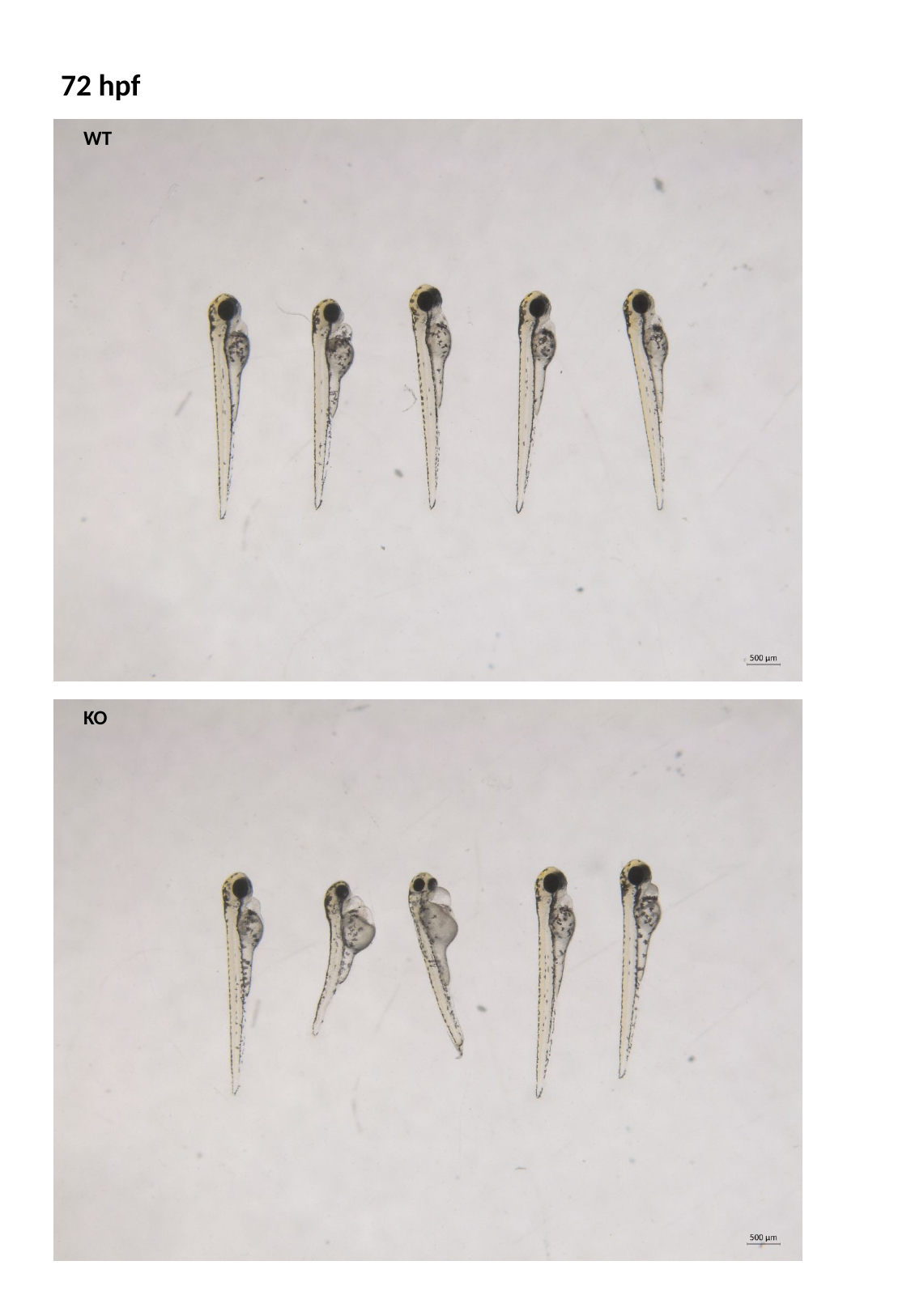

72 hpf
WT
KO

## Slide 4
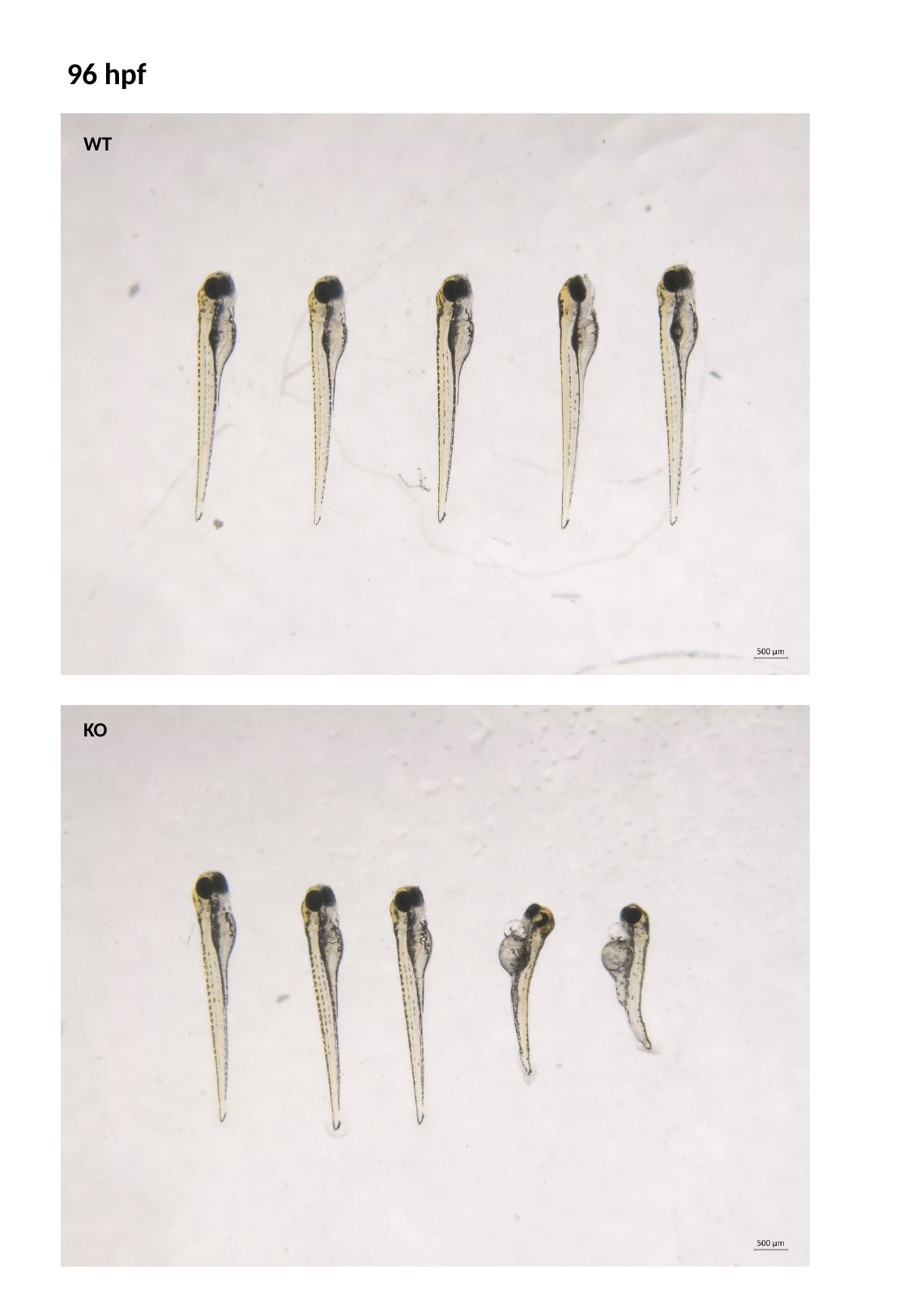

96 hpf
WT
KO

## Slide 5
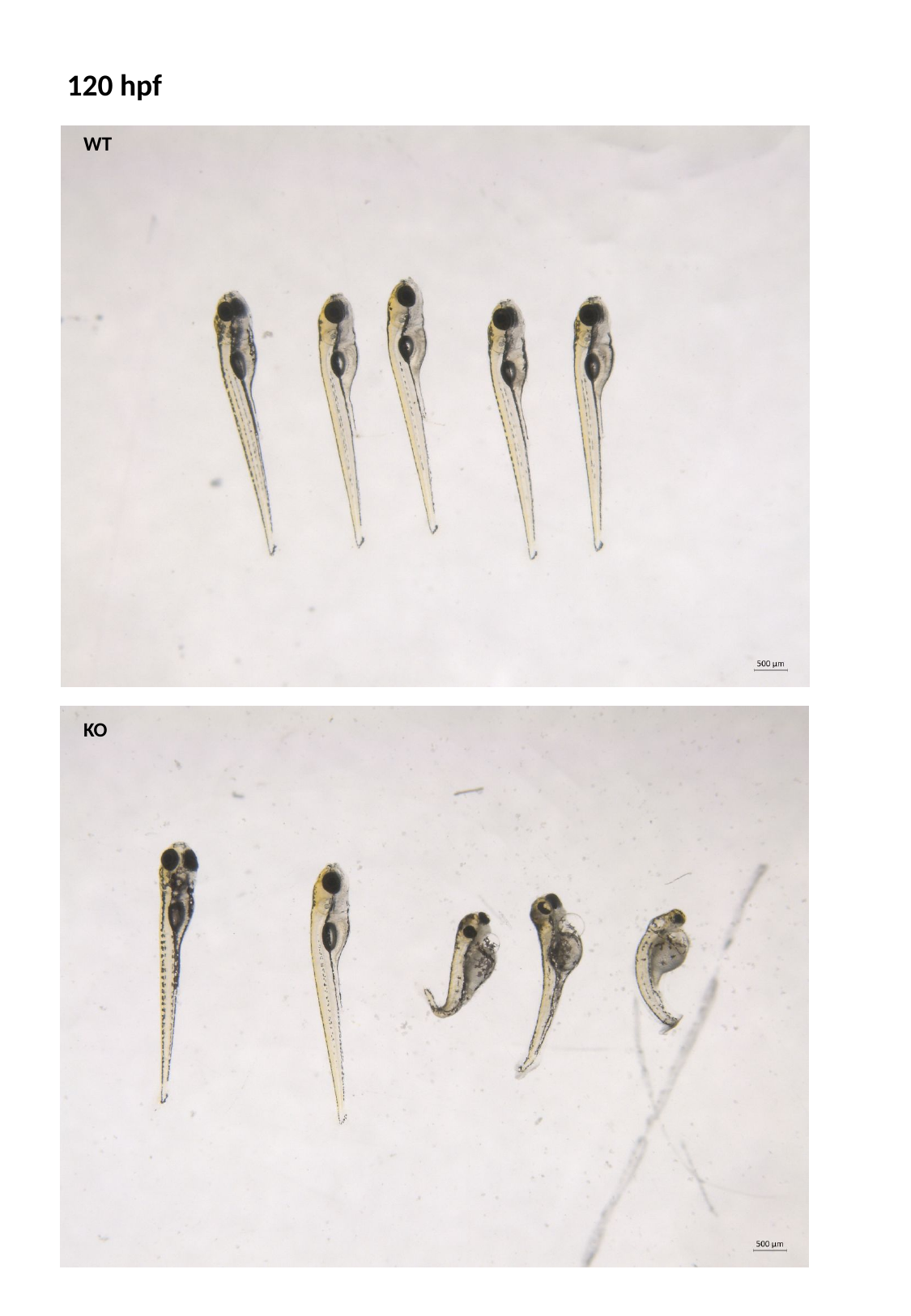

120 hpf
WT
KO

## Slide 6
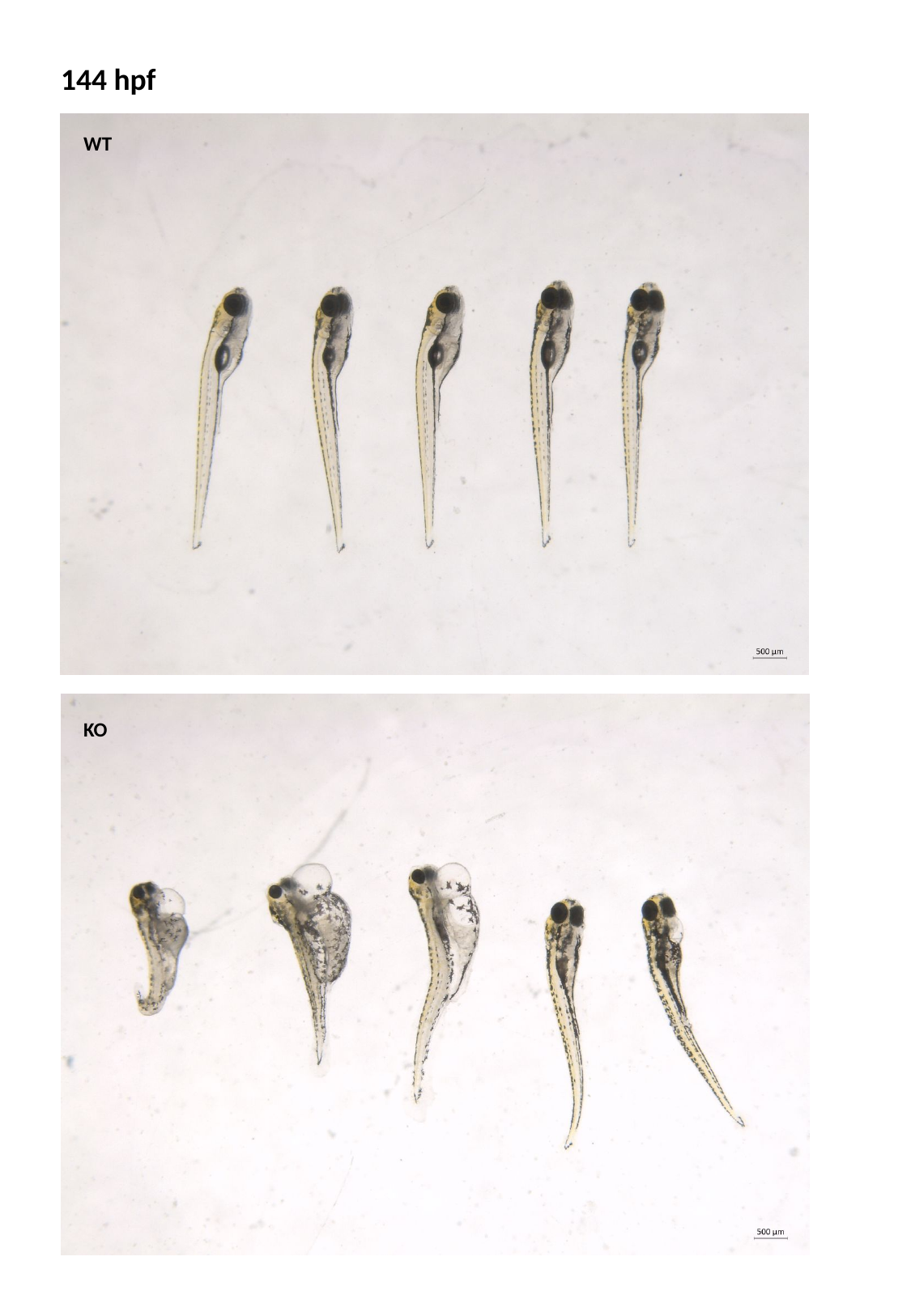

144 hpf
WT
KO

Supplement: Supplementary file 3 — Additional File 3. Images of embryos from all stages, views, and genotypes. The images were obtained using an M205C stereomicroscope (Leica Microsystems) at 10x magnification with embryos anesthetized with 0.4% tricaine. (PPTX 20745 kb) [file 12864_2022_8369_MOESM3_ESM.pptx]
